# Identifying 4 Novel lncRNAs as Potential Biomarkers for Acute Rejection and Graft Loss of Renal Allograft

**DOI:** 10.1155/2020/2415374

**Published:** 2020-11-28

**Authors:** Zedan Zhang, Yanlin Tang, Hongkai Zhuang, Enyu Lin, Lu Xie, Xiaoqiang Feng, Jiayi Zeng, Yanjun Liu, Jiumin Liu, Yuming Yu

**Affiliations:** ^1^Department of Urology, Guangdong Provincial People's Hospital, Guangdong Academy of Medical Sciences, Guangzhou 510080, China; ^2^Shantou University Medical College, Shantou 515041, China; ^3^Department of Immunology, School of Basic Medical Science, Southern Medical University, Guangzhou, China

## Abstract

Acute rejection (AR) after kidney transplant is one of the major obstacles to obtain ideal graft survival. Reliable molecular biomarkers for AR and renal allograft loss are lacking. This study was performed to identify novel long noncoding RNAs (lncRNAs) for diagnosing AR and predicting the risk of graft loss. The several microarray datasets with AR and nonrejection specimens of renal allograft downloaded from Gene Expression Omnibus database were analyzed to screen differentially expressed lncRNAs (DElncRNAs) and mRNAs (DEmRNAs). Univariate and multivariate Cox regression analyses were used to identify optimal prognosis-related DElncRNAs for constructing a risk score model. 39 common DElncRNAs and 185 common DEmRNAs were identified to construct a lncRNA-mRNA regulatory relationship network. DElncRNAs were revealed to regulate immune cell activation and proliferation. Then, 4 optimal DElncRNAs, ATP1A1-AS1, CTD-3080P12.3, EMX2OS, and LINC00645, were selected from 17 prognostic DElncRNAs to establish the 4-lncRNA risk score model. In the training set, the high-risk patients were more inclined to graft loss than the low-risk patients. Time-dependent receiver operating characteristics analysis revealed the model had good sensitivity and specificity in prediction of 1-, 2-, and 3-year graft survival after biopsy (AUC = 0.891, 0.836, and 0.733, respectively). The internal testing set verified the result well. Gene set enrichment analysis which expounded NOD-like receptor, the Toll-like receptor signaling pathways, and other else playing important role in immune response was enriched by the 4 lncRNAs. Allograft-infiltrating immune cells analysis elucidated the expression of 4 lncRNAs correlated with gamma delta T cells and eosinophils, etc. Our study identified 4 novel lncRNAs as potential biomarkers for AR of renal allograft and constructed a lncRNA-based model for predicting the risk of graft loss, which would provide new insights into mechanisms of AR.

## 1. Introduction

Kidney transplant is increasingly and widely acceptable treatment for end-stage renal disease. However, acute rejection (AR) that occurs days to months after renal transplantation is one of the major obstacles to obtain ideal long-term graft survival, while plenty of patients with end-stage renal disease are still waiting for renal grafts [1–3]. Therefore, it is important to routinely monitor the function of allograft and diagnose AR promptly. The serum creatinine as a regular biomarker for inferring AR can be late for the change of allograft condition, while kidney biopsy, regarded as the gold standard for diagnosing AR, still has limitations, such as potentially variable artificial diagnosis and others, which reflect that it requires additional molecular biomarkers to become a unified diagnostic criteria [4, 5]. Although the rate of acute rejection has drastically decreased over the past five decades due to the advances in immunosuppressive therapy, some evidence revealed that improvements of traditional immunosuppression in transplant recipients for preventing AR could improve the early outcome of renal allograft but not the long-term graft survival [1, 6]. Moreover, high-dose immunosuppressants such as steroids and/or lymphocyte-depleting antibodies enhance the risk of infection, sepsis, and even cancer, potentially bringing about death of patients with functioning allograft [7–9]. Hence, it is necessary to identify novel biomarkers for AR of renal allograft, especially prognosis-related biomarkers, which probably make available for speculating the underling mechanisms of AR and provide druggable targets in the future.

Long noncoding RNAs (lncRNAs) defined as more than 200 nucleotides RNA without the ability of protein coding were widely investigated and found to affect development of human diseases through regulating neighbouring genes and chromatin topology, scaffolding, and decoying proteins [10, 11]. Their effective roles have been recognized in renal transplantation, renal ischemia, fibrosis, injury, inflammation, glomerular diseases, and renal cell carcinoma [12]. Chen et al. [13] and Zou et al. [14] identified dysregulated lncRNAs in AR specimens when compared with nonrejection specimens. Xu et al. [15] also screened the lncRNAs associated with chronic damage and graft loss after renal transplantation. Besides, lncRNA RP11-354P17.15-001 in urine was reported that it might serve as a novel biomarker for AR of kidney [16]. However, the underlying mechanism through which lncRNAs contribute the rejection or injury is rarely known. Atianand et al. has discovered that lncRNAs take part in development and differentiation of innate and adaptive immune cells [17]. Meanwhile, some studies revealed that allograft-infiltrating immune cells own potential diagnostic and prognostic values in AR after renal transplantation [18, 19], which turns out to be a major target for immunosuppression therapy [20]. To the best of our knowledge, no exact lncRNA has been regarded as the known criteria to help diagnose AR and predict the prognosis of graft survival so far. Thus, lncRNAs related to AR of kidney transplant, graft survival, and allograft-infiltrating immune cells were screened by our study based on the public datasets. This will probably help develop novel biomarkers for AR of renal allograft, directly or indirectly impact current clinical practices, and provide new insights into the mechanism of AR.

## 2. Material and Methods

### 2.1. GEO Dataset Acquisition and Preprocessing

Gene Expression Omnibus database (GEO, https://www.ncbi.nlm.nih.gov/geo/) is a public repository of high-throughput gene expression data at the National Center of Biotechnology Information, which is submitted by researchers in various fields, and the data are freely accessible. Five datasets, GSE34437 [21], GSE75693 [22], GSE50058 [23], GSE76882 [24], and GSE21374 [25], containing RNA sequencing data extracted from kidney biopsies from kidney transplant patients with different graft state were selected. Their series matrix and MINiML formatted family files were downloaded. Two platforms were identified: GSE76882 dataset was from Affymetrix HT HG-U133+ PM Array Plate, while others were all from Affymetrix Human Genome U133 Plus 2.0 Array. The data tables of the two platforms were also downloaded to obtain the gene symbol for reannotating the Affymetrix Probe Set ID in the series matrix files. Besides, we extracted the corresponding clinical information from the MINiML formatted family files using SangerBox tool (Version 1.0.9, http://sangerbox.com/). Among these datasets, the diagnosis criteria of AR for allograft were biopsy-proven based on the Banff criteria scored by blinded pathologists, which accompanied rising serum creatinine. Nonrejection or stable biopsies were regarded as stable graft function and absence of significant pathological injury based on the Banff criteria. As for the protocol surveillance or for-cause biopsies, the GSE34437, GSE50058, and GSE76882 datasets contained mixed samples, while the GSE75693 and GSE21374 datasets embrace for-cause biopsies. In our study, biopsy-proven acute rejection, borderline acute rejection, and acute rejection with interstitial fibrosis and tubular atrophy were incorporated into the AR group in our study. Specimens diagnosed with BK virus nephritis in the datasets were excluded. The number of patients with different graft state and outcome in the five datasets is shown in Table 1. The more detailed clinical information of the kidney biopsies is given in Tables S1 and S2.

Therefore, GSE34437 and GSE75693 datasets were merged into a single cohort, in the following called the merged dataset, due to the small number of specimens and followed by batch normalization using “sva” R package to eliminate the batch effect. Then in the merged dataset, GSE50058 and GSE76882 datasets were used for differential expression analysis. The time from biopsy to the graft failure or patient censored in GSE21374 dataset was defined as graft survival, which was used for subsequent graft survival analysis of the screened genes. The 282 patients with information of graft survival in GSE21374 dataset were randomly divided into the training set (*n* = 198) and the testing set (*n* = 84) by a ratio of 7 to 3 using “caret” R package.

### 2.2. Differential Expression Analyses of lncRNAs and mRNAs

The expression data of GEO datasets were checked whether they were standardized and were quantile normalized using the normalizeBetweenArrays function from “limma” R package (Figure S1). Then, lncRNAs and protein-coding genes were extracted from the matrix based on the human genome (CRCh38) from database Ensembl (http://asia.ensembl.org/index.html). Moreover, the gene was excluded if it is an unrecognized gene or the sum of expression level for each specimen is less than 1. Finally, 1236 lncRNAs and 1234 lncRNAs were obtained from the merged dataset and GSE50058 datasets, respectively. 16413, 16432, and 14064 protein-coding genes were obtained from the merged datasets GSE50058 and GSE76882, respectively.

The “limma” R package was used to screen differentially expressed lncRNAs (DElncRNAs) and differentially expressed mRNAs (DEmRNAs) between kidney transplant patients with AR and stable function. Then, the DElncRNAs from the merged dataset and GSE50058 dataset were intersected to obtain common DElncRNAs, while the DEmRNAs from the merged dataset, GSE50058 and GSE76882 were intersected to obtain common DEmRNAs for subsequent analyses. Adjusted *p* value < 0.05 and absolute log_2_ fold change (FC) > 0.5 were set to screen DElncRNAs. Adjusted *p* value < 0.05 and absolute log_2_ fold change (FC) > 1 were used to screen DEmRNAs.

### 2.3. Construction of lncRNA-mRNA Regulatory Relationship Network

To elucidate the expressional relationship between dysregulated lncRNAs and mRNAs in AR of kidney allograft, the Pearson correlation analysis was performed with the cutoff criteria of absolute coefficient of correlation (*r*) > 0.7 and *p* value < 0.01. The visualization of the regulatory network was constructed using Cytoscape (V3.7.2, https://cytoscape.org/).

### 2.4. Functional Annotation and Enrichment Analyses of DElncRNAs

Gene Ontology (GO) and Kyoto Encyclopedia of Genes and Genomes (KEGG) enrichment analyses were conducted using “clusterProfiler” R package for revealing the potential gene function terms and enriched signaling pathways of the DEmRNAs in the lncRNA-mRNA regulatory relationship network, which could indirectly indicate the potential biological function of DElncRNAs. GO enrichment analysis contains the aspects of molecular function (MF), cellular components (CC), and biological processes (BP). Before doing the enrichment analyses, gene symbol was converted to Entrez Gene IDs using “http://org.hs.eg.db” R package in order to the mapping between GO or KEGG terms and Entrez Gene IDs. The cutoff value for the enrichment analyses was adjusted *p* value < 0.05.

### 2.5. Identification of Prognostic lncRNAs

The normalized expression data of DElncRNAs and corresponding survival time of grafts from the training set (*n* = 198) in GSE21374 dataset were used to identify the prognosis-related DElncRNAs using univariate Cox proportional hazards regression analysis. Then, the patients were divided into two groups, high- and low-expression groups by the median expression level as a cutpoint of each prognostic DElncRNA, which was determined by “survminer” R package. Then, “survival” R package was performed to implement a log-rank test and draw Kaplan-Meier curves to compare the graft survival rate between high- and low-expression levels of the prognostic DElncRNAs [26]. Afterwards, the prognostic DElncRNAs screened from the univariate Cox proportional hazard regression were enrolled in stepwise multivariate Cox proportional hazard regression method to select the optimal gene model with the minimum AIC value. A *p* value < 0.05 was considered as significant.

### 2.6. Establishment and Assessment of Multi-lncRNA Model

The final optimal prognosis-related DElncRNAs screened from multivariate Cox regression were used to construct a risk score model to evaluate graft survival in renal transplant patients who were diagnosed with acute rejection. The risk score of each patient was calculated by multiplication of the regression coefficient of the lncRNAs obtained from multivariate Cox regression and their expression level and finally summing, which was the following:

(1) $$\begin{array}{c} \text{Risk score}=\overset{n}{\underset{i=1}{\sum }}\beta_{i}*{\text{Exp}}_{i}, \end{array}$$

where *n*, *β*, and Exp are the number, the regression coefficient, and the expression value of the prognosis-related DElncRNAs, respectively.

Then, the median risk score was regarded as the cutoff point to stratify the patients into high-risk and low-risk groups in the training set. The Kaplan-Meier (KM) survival analysis was performed to compare the graft survival between these two groups. Moreover, the time-dependent receiver operating characteristics (tROC) curve was used to evaluate the specificity and sensitivity of the graft survival prediction using “survivalROC” R package.

In order to assess the prediction value of the model, the testing set (*n* = 84) in GSE21374 dataset was used to perform validation. The risk score of each renal transplant patient was calculated through the above formula with the same coefficients used in the training set. Then, the patients were stratified into high-risk and low-risk groups using the same cutoff point in the training set. Besides, we used KM survival analysis and tROC analysis to validate the model in the testing set.

### 2.7. Immune Cell Infiltration Analysis and Gene Set Enrichment Analysis of Final Optimal lncRNAs

CIBERSORT (cell type identification by estimating relative subsets of RNA transcripts, https://cibersort.stanford.edu/) [27] is a computational approach for characterizing cell composition of complex tissues including fresh, frozen, and fixed tissues from the gene expression profiles. It outperformed many other estimating methods with respect to noise and unknown mixture content [28]. A signature matrix of the collated 547-gene expression datasets associated with 22 immune cell types was downloaded from CIBERSORT web portal, which encompasses T cells, B cells, natural killer cells, dendritic cells, eosinophils, etc. Then, the significantly different proportions of infiltrating immune cells in the merged dataset and GSE76882 dataset between acute rejection and nonrejection specimens were calculated using the CIBERSORT algorithm and Wilcoxon method for variance by R software with the criteria of *p* value < 0.05. The correlation between common infiltrating immune cells and expression of the final optimal lncRNAs was further analyzed using Pearson correlation analysis, which considered ∣*r* | >0.3 and *p* value < 0.01 as significant.

For identifying the biological pathways of the final optimal lncRNAs, gene set enrichment analysis (GSEA) [29] was performed on JAVA 8.0 platform using GSEA software (V4.0.4, http://software.broadinstitute.org/gsea/). The median expression of the lncRNAs was regarded as a cutoff point to divide all the samples into high- and low-expression groups. Then, the annotated gene sets “c2.cp.kegg.v7.0.symbols” and “c5.all.v7.0.symbols” downloaded from the Molecular Signatures Database (MSigDB) were adopted as the reference gene set to calculate enrichment score (ES). The number of permutations was 1000. Gene size < 15 or >500 was excluded. The enrichment pathways with ∣ES | >1.5, normalized *p* value < 0.05, and FDR < 0.25 were expected as significant [29].

### 2.8. Statistical Analysis

R software (V3.6.1, The R Foundation for Statistical Computing, 2019) was used to perform all statistical analysis. Volcano plots of DElncRNAs and DEmRNAs were plotted using “ggrepel” R package, while heat maps of DElncRNAs and DEmRNAs were plotted using “pheatmap” R package with zero-mean normalization. The expressional boxplots between the AR and NR groups were analyzed using the Mann–Whitney *U* test. The boxplots and correlation graphs were drawn by Prism 8 (GraphPad). To validate the association between the final optimal lncRNAs and AR of renal allograft, logistic regression analysis was conducted and area under the ROC curve (AUC) was also calculated to illustrate their diagnostic accuracy. For Kaplan-Meier curves, *p* values and hazard ratio (HR) with 95% confidence interval (CI) were generated by the log-rank tests and Cox regression methods. All statistical tests were two-sided. *p* value < 0.05 was considered as statistically significant.

## 3. Results

### 3.1. Identification of DElncRNAs and DEmRNAs in AR

**Figure 1:**
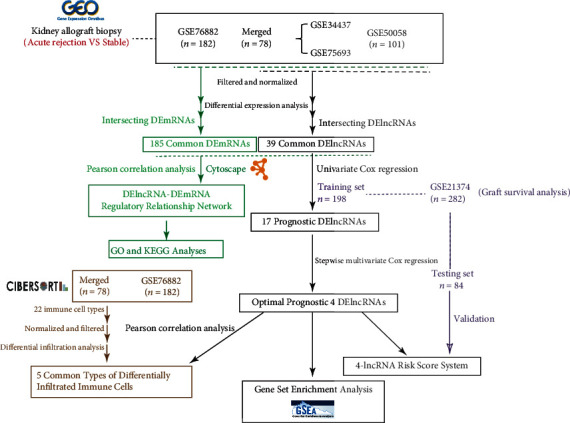
Flowchart of our study design.

The flow chart of our whole study is shown in Figure 1.

**Figure 2:**
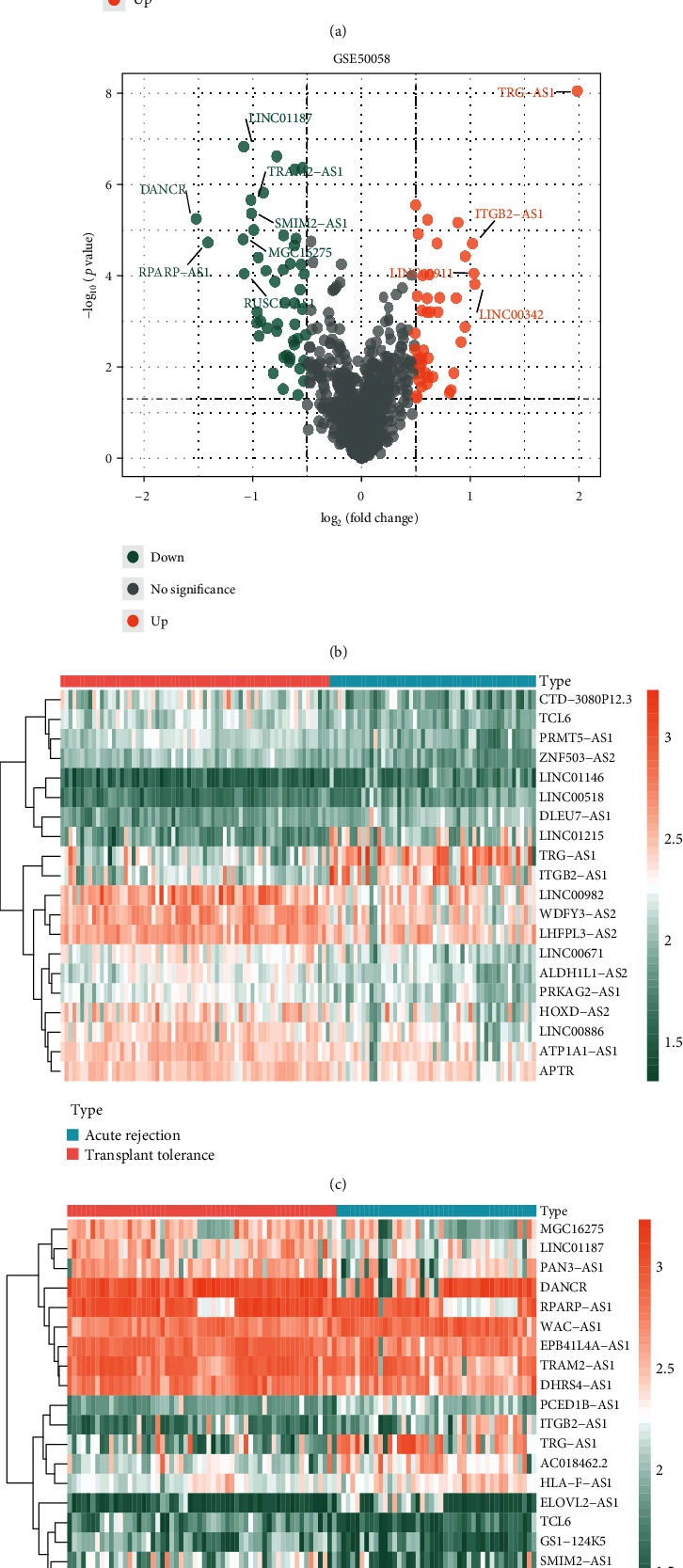
Identification of DElncRNAs and DEmRNAs (adjusted *p* value < 0.05 and ∣log_2_FC | >0.5 for DElncRNAs; adjusted *p* value < 0.05 and ∣log_2_FC | >1 for DEmRNAs). (a, b) Volcano plots of DElncRNAs in the merged dataset and GSE50058 dataset, respectively. (c, d) Heat maps of top 20 DElncRNAs in the merged dataset and GSE50058 dataset, respectively. (e) Venn diagram of 39 common DElncRNAs between the merged dataset and GSE50058 dataset. (f) Venn diagram of 185 common DEmRNAs among the merged dataset, GSE50058 dataset, and GSE76882 dataset (volcano plots and heat maps of DEmRNAs in these three datasets are presented in Figure S2). DElncRNAs: differentially expressed lncRNAs; DEmRNAs: differentially expressed mRNAs; FC: fold change.

After differential expression analysis via limma R package, there were 134 DElncRNAs and 553 DEmRNAs in the merged dataset; 63 DElncRNAs and 439 DEmRNAs in GSE50058 dataset; and 620 DEmRNAs in GSE76882. The cluster heat maps of top 20 DElncRNAs and volcano plots of DElncRNAs in the merged dataset and GSE50058 dataset are shown in Figures 2(a)–2(d). Then, the common 39 DElncRNAs and 185 DEmRNAs were obtained via integration using “vennDiagram” R package, which are shown in Figures 2(e) and 2(f).

### 3.2. lncRNA-mRNA Regulatory Relationship Network Construction and Functional Enrichment Analysis

**Figure 3:**
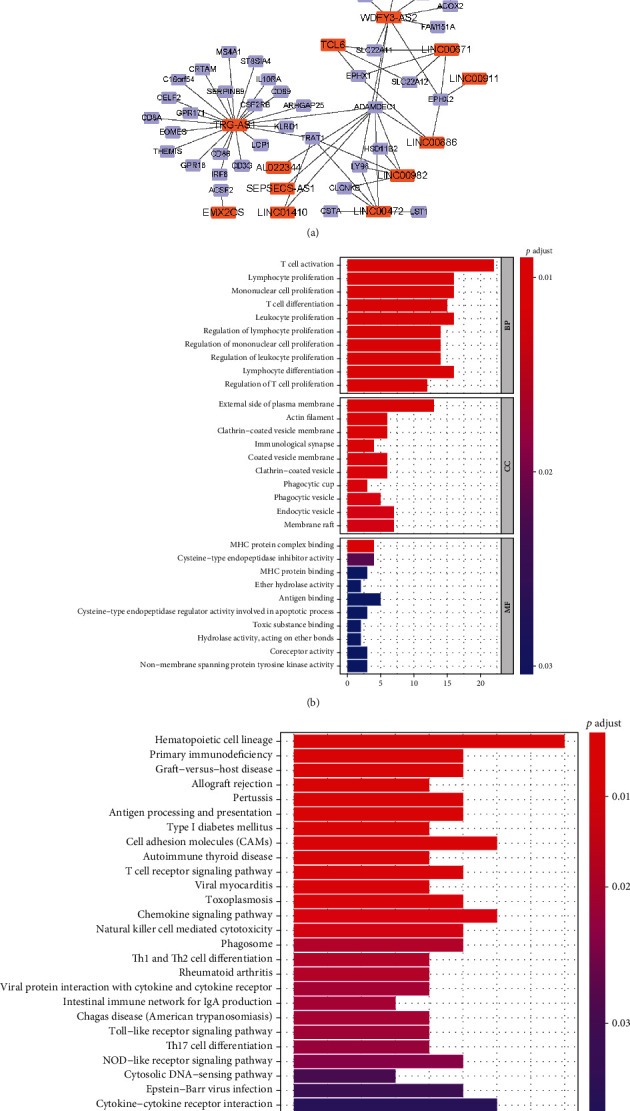
Relationship between DElncRNAs and DEmRNAs and corresponding functional enrichment analysis of DEmRNAs. (a) The DElncRNA-DEmRNA regulatory relationship network. The orange nodes represent the common DElncRNAs. The purple nodes represent the DEmRNAs correlated with the DElncRNAs. The relationship is shown based on Pearson correlation analysis with the criteria of ∣*r* | >0.7 and *p* value < 0.05. (b) GO functional enrichment analysis of DEmRNAs involved in the network. (c) KEGG functional enrichment analysis of DEmRNAs involved in the network. DElncRNAs: differentially expressed lncRNAs; DEmRNAs: differentially expressed mRNAs; GO: Gene Ontology; KEGG: Kyoto Encyclopedia of Genes and Genomes.

For identifying the coexpression relationship between DElncRNAs and DEmRNAs in AR of renal allograft, the Pearson correlation analysis was performed based on GSE50058 dataset and totally there were 101 lncRNA-mRNA pairs with positive correlation and 16 lncRNA-mRNA pairs with negative correlation (∣*r* | >0.7, *p* value < 0.05). The relevant positive and negative correlation networks were constructed using Cytoscape and they are shown in Figures 3(a) and 3(b). Besides, in order to figure out the potential biological function of common DElncRNAs, the coexpression DEmRNAs were used for GO and KEGG analyses. The significantly enriched GO terms are shown in Figure 3(c). Top 10 enriched terms in BP, CC, and MF are listed in the figure. In BP category, most of the enriched terms were associated with regulation of immune cell activation and proliferation. In CC and MF category, “external side of plasma membrane” and “G protein-coupled receptor binding” were the most enriched terms, respectively. The significantly enriched KEGG pathways are shown in Figure 3(d). “Cytokine-cytokine receptor interaction” was the most enriched pathway. “Allograft rejection” and “MHC protein complex binding” were also enriched which demonstrated the good reliability of the differential expression analysis screened by our study. Moreover, signaling pathways like “NOD-like receptor signaling pathway” and “Toll-like receptor signaling pathway” were enriched. Pathways involved in “Allograft rejection” and “Graft-versus-host disease” were enriched as well.

### 3.3. Screening of Prognosis-Related DElncRNAs

The graft survival data after biopsy of renal allograft and the expression data of 39 common DElncRNAs in the training set were subjected to univariate Cox proportional hazards regression analysis with the significant threshold of *p* value < 0.05. Therefore, 15 DElncRNAs associated with the graft survival were identified, among which there were 2 positive DElncRNAs (HR > 1) and 13 negative DElncRNAs (HR < 1). Then, these 17 DElncRNAs were used in subsequent stepwise multivariate Cox proportional hazards regression analysis. Finally, the optimal 4-lncRNA combination was obtained with the minimum Akaike's information criterion value (AIC = 317.29), which contained ATP1A1-AS1, CTD-3080P12.3, EMX2OS, and LINC00645. The results of Cox regression analysis are shown in Table 2.

### 3.4. Expression Profiles and Survival Analysis of the Optimal 4 lncRNAs

**Figure 4:**
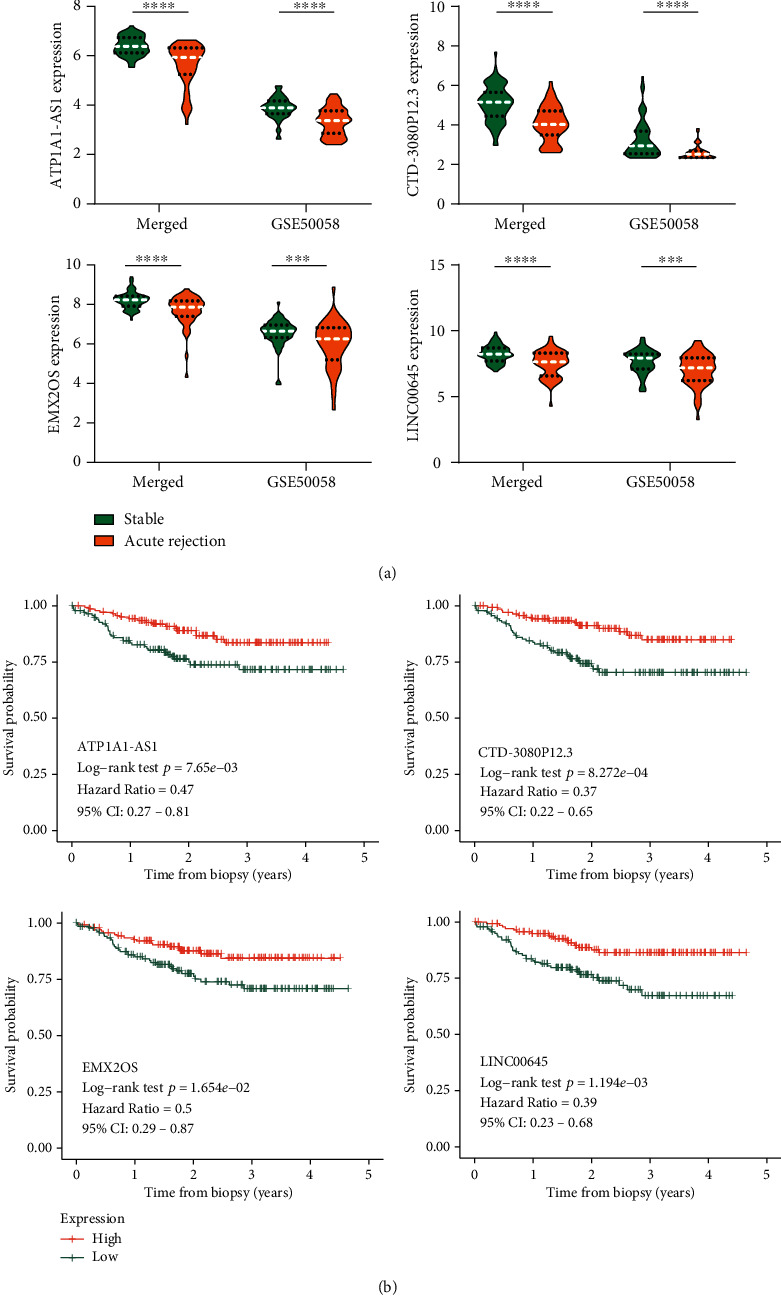
Expression pattern and Kaplan-Meier survival analysis of the optimal 4 lncRNAs. (a) The expression pattern of ATP1A1-AS1, CTD-3080P12.3, EMX2OS, and LINC00645 in the merged dataset and GSE50058 dataset. (b) Graft survival analysis of ATP1A1-AS1, CTD-3080P12.3, EMX2OS, and LINC00645 in the patients of GSE21374 datasets. ^∗^*p* < 0.05, ^∗∗^*p* < 0.01, ^∗∗∗^*p* < 0.001, and ^∗∗∗∗^*p* < 0.0001.

The expression profiles of the optimal 4 DElncRNAs between AR and NR specimens in the merged dataset and GSE50058 dataset are presented in Figure 4(a), which indicated that the 4 DElncRNAs were significantly downregulated in AR of renal allograft (*p* < 0.001). Moreover, the result of logistic regression analysis based on the merged dataset and GSE50058 dataset verified that the 4 DElncRNAs were significantly associated with the AR of renal allograft (*p* < 0.01) (Table 3).

In the meanwhile, ROC analysis was performed as well to obtain their AUC values and standard error. In the merged dataset, AUC_ATP1A1‐AS1_ = 0.863 (0.041), AUC_CTD‐3080P12.3_ = 0.852 (0.044), AUC_EMX20S_ = 0.750 (0.060), and AUC_LINC00645_ = 0.756 (0.059). In GSE50058 dataset, AUC_ATP1A1‐AS1_ = 0.780 (0.048), AUC_CTD‐3080P12.3_ = 0.744 (0.049), AUC_EMX20S_ = 0.666 (0.057), and AUC_LINC00645_ = 0.718 (0.052).

KM survival curves were plotted to evaluate the prognostic value of the 4 DElncRNAs (Figure 4(b)), in which the median expression value of each DElncRNA was regarded as a cutoff point to partition the patients into the high-expression and low-expression groups. Low expression of the 4 DElncRNAs was associated with the poor prognosis of the renal allograft. The KM survival curves of other 11 prognosis-related DElncRNAs screened from univariate Cox regression analysis are also shown in Figure S3.

### 3.5. Construction and Validation of the 4-lncRNA Model

The Cox coefficients of the 4 lncRNAs obtained from the multivariate Cox proportional hazards regression analysis were used to multiply their expression values for calculating the risk score of each patient in GSE21374 dataset. Risk score = (−1.9663)∗Exp_(ATP1A1‐AS1)_ + (−2.28396)∗Exp_(CTD‐3080P12.3)_ + (−1.12712)∗Exp_(EMX20S)_ + (−0.59929)∗Exp_(LINC00645)_.

**Figure 5:**
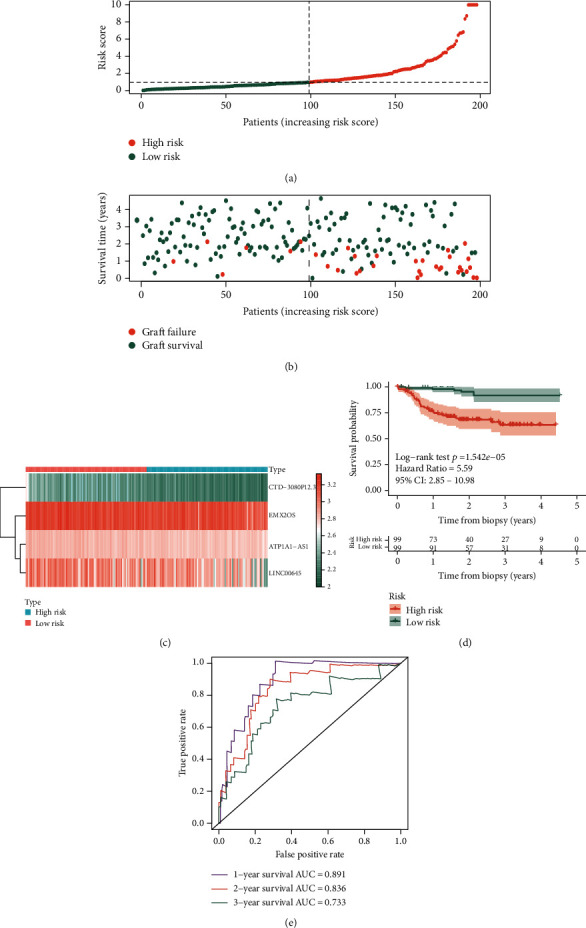
Prognostic analysis of the 4-lncRNA risk score in the training set of GSE21374 dataset. (a) The curve of risk score. The dotted line represents the cutoff score which divides the patients into the low-risk group and high-risk group. (b) The survival status of renal allograft. The coral dots represent graft failure while the aquamarine dots represent graft survival. (c) Heat map of the 4-lncRNA expression profiles in the low-risk group and high-risk group. (d) KM survival analysis of the risk score model. (e) Time-dependent ROC analysis of the risk score model. KM: Kaplan-Meier; ROC: receiver operating characteristic.

Then, the patients in the training set were divided into high- and low-risk groups based on the median risk score determined by “survminer” R package, in which the risk score curve is shown in Figure 5(a). The corresponding graft survival status of the patients in the training set is demonstrated in Figure 5(b), which suggests that there were more patients who were classified as high risk tend to get graft failure. The expression profiles of the 4 DElncRNAs in AR and stable specimens are visualized in the cluster heat map (Figure 5(c)). The KM survival curve revealed that the high-risk group patients have worse graft survival outcome than that of the low-risk group patients (*p* < 0.001) (Figure 5(d)). Moreover, tROC analysis was performed and AUC values of 1-, 2-, and 3-year graft survival after biopsy are 0.891, 0.836, and 0.733 (Figure 5(e)).

**Figure 6:**
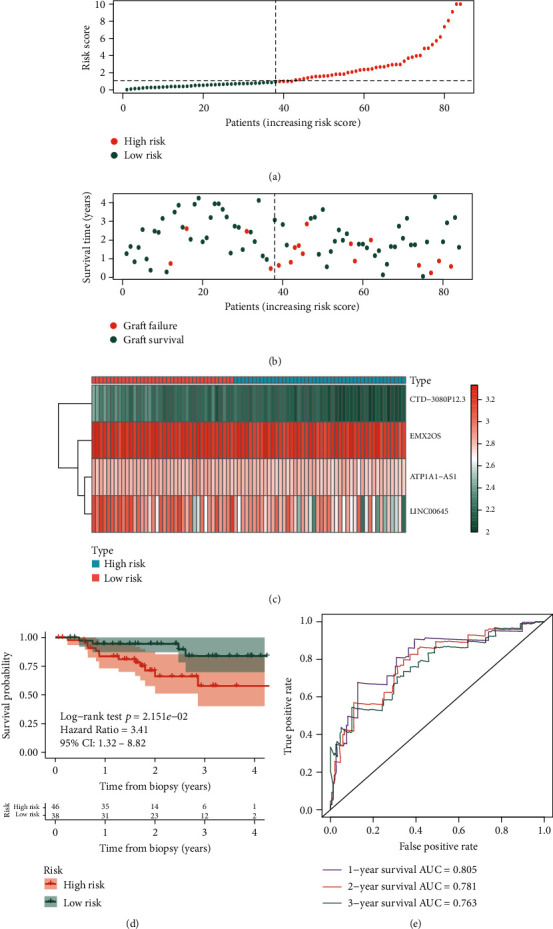
Prognostic analysis of the 4-lncRNA risk score in the testing set of GSE21374 dataset. (a) The curve of risk score. The dotted line represents the cutoff score which divides the patients into the low-risk group and high-risk group. (b) The survival status of renal allograft. The coral dots represent graft failure while the aquamarine dots represent graft survival. (c) Heat map of the 4-lncRNA expression profiles in the low-risk group and high-risk group. (d) KM survival analysis of the risk score model. (e) Time-dependent ROC analysis of the risk score model. KM: Kaplan-Meier; ROC: receiver operating characteristic.

In order to verify the results obtained from the training set, the testing set was used for the same analysis. The cutoff point of the risk score in the training set was used to divide the patients into high- and low-risk groups in the testing set (Figure 6(a)), and more patients got graft failure in the high-risk groups (Figure 6(b)). The cluster heat map of the 4 DElncRNAs is also presented in Figure 6(c). The KM survival curves verified the significant difference of graft survival between the high- and low-risk groups (*p* < 0.05) (Figure 6(d)). AUC values of 1-, 2-, and 3-year graft survival after biopsy in the testing set are 0.805, 0.781, and 0.763 (Figure 6(e)).

### 3.6. Correlation between the Optimal 4 lncRNAs and Immune Cell Infiltration

For determining the types of infiltrating immune cells involved in AR of renal allograft, CIBERSORT algorithm was performed to speculate the percentage of 22 types of immune cells in allograft specimens. After the calculation and filter with the criteria of *p* value < 0.05, the merged dataset and GSE76882 contained 68 and 108 AR and stable specimens, while GSE50058 contained only 15 AR and stable specimens. Therefore, the GSE50058 dataset was excluded in the comparison analysis for revealing significant different infiltrated immune cells between AR and stable specimens.

**Figure 7:**
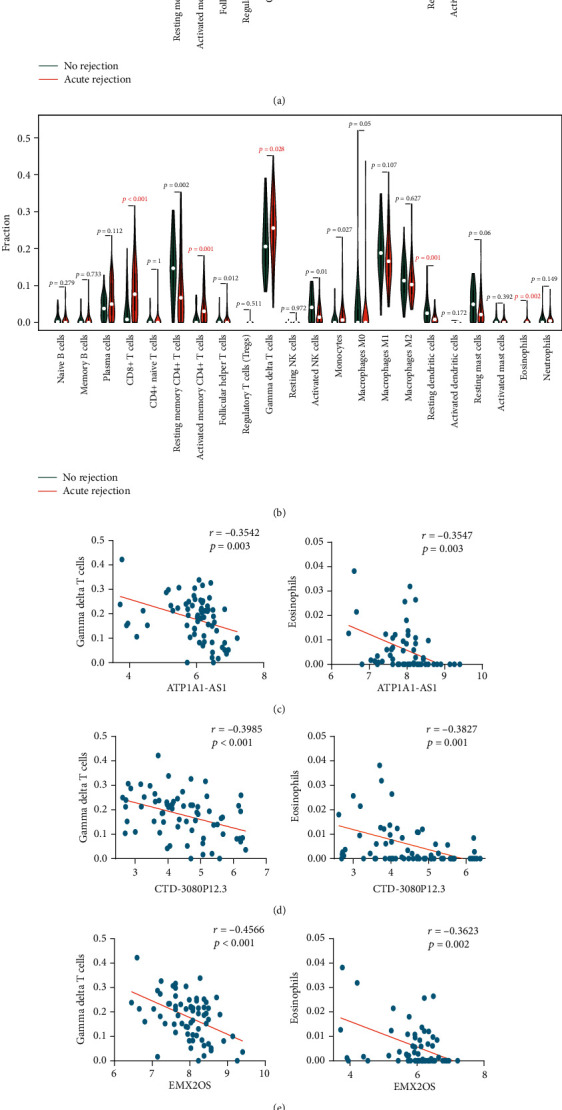
Pearson correlation analysis between the 4 lncRNAs and 5 common different allograft-infiltrating immune cell types. (a, b) CIBERSORT estimation of 22 immune cell types between AR and nonrejection specimens in the merged dataset and GSE76882 dataset, respectively. CD8^+^ T cells, activated memory CD4^+^ T cells, gamma delta T cells, resting dendritic cells, and eosinophils are 5 significantly different immune cell types common to the merged dataset and GSE76882 dataset. (c) The correlation between the expression of ATP1A1-AS1 and gamma delta T cells as well as eosinophils. (d) The correlation between the expression of CTD-3080P12.3 and gamma delta T cells as well as eosinophils. (e) The correlation between the expression of EMX2OS and gamma delta T cells as well as eosinophils. (f) The correlation between the expression of LINC00645 and CD8^+^ T cells as well as resting dendritic cells. CIBERSORT: cell type identification by estimating relative subsets of RNA transcripts; AR: acute rejection.

The comparison of immune cell infiltration between AR and stable specimens in the merged dataset and GSE76882 dataset is shown in Figures 7(a) and 7(b), respectively. In these two datasets, there were 5 common significantly different immune cell types: CD8^+^ T cells (*p* = 0.018), activated memory CD4^+^ T cells (*p* = 0.001), gamma delta T cells (*p* = 0.028), and eosinophils (*p* = 0.011) were more infiltrated in AR specimens, while resting dendritic cells (*p* = 0.005) was less infiltrated in AR specimens compared with stable specimens. Then, the correlation between the optimal 4 lncRNAs and the 5 common significantly different immune cell types is explored and presented in Figures 7(c)–7(f) based on the merged dataset. The expressions of ATP1A1-AS1, CTD-3080P12.3, and EMX2OS were all negatively correlated with the gamma delta T cells and eosinophils. The expression of LINC00645 was negatively correlated with CD8^+^ T cells and positively correlated with resting dendritic cells.

### 3.7. Gene Set Enrichment Analysis

**Figure 8:**
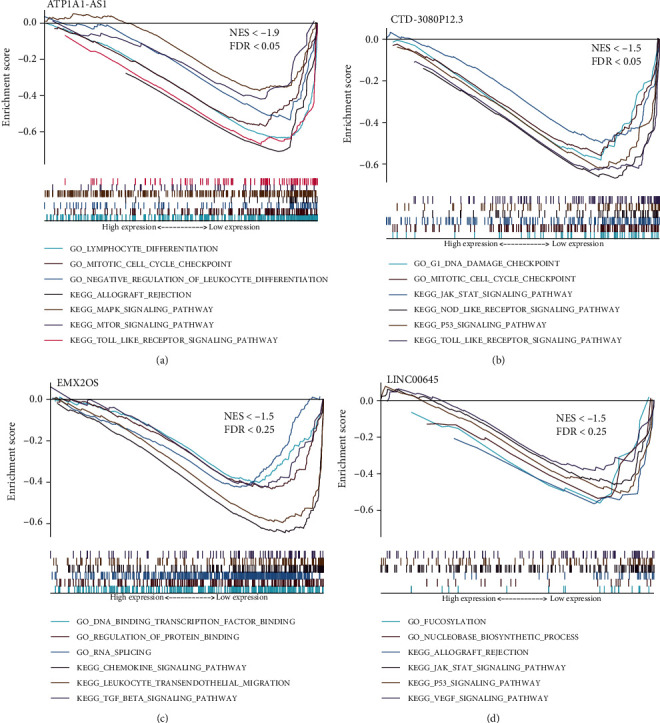
GSEA of the 4 lncRNAs. (a) ATP1A1-AS1. (b) CTD-3080P12.3. (c) EMX2OS. (d) LINC00645. GSEA: gene set enrichment analysis.

The potential biological functions of ATP1A1-AS1, CTD-3080P12.3, EMX2OS, and LINC00645 were mined using GSEA. As the results shown in Figure 8(a), lymphocyte differentiation and mitotic cell cycle checkpoint gene sets were found to be enriched in the low expression of ATP1A1-AS1. G1 DNA damage checkpoint and mitotic cell cycle checkpoint gene sets were enriched in the low expression of CTD-3080P12.3 (Figure 8(b)). For the low expression of EMX2OS, DNA-binding transcription factor and RNA splicing gene sets were enriched (Figure 8(c)). Fucosylation and nucleobase biosynthetic process gene sets were enriched in the low expression of LINC00645 (Figure 8(d)). Moreover, several important signaling pathways which play important roles in allograft rejection were found to enriched by the low expression of these 4 lncRNAs, such as the MAPK signaling pathway, MTOR signaling pathway, Toll-like receptor signaling pathway, NOD-like receptor signaling pathway, p53 signaling pathway, TGF-*β* signaling pathway, JAK-STAT signaling pathway, and VEGF signaling pathway, where the findings provided good clues for exploring the potential specific function of these four lncRNAs in AR of renal allografts.

## 4. Discussion

Mining high-throughput microarray datasets which contain thousands of genes in small sample sizes are the cost-effective manner in exploring the fields of genetic characteristics and functional genomics [30]. lncRNAs as noncoding RNAs have been reported that they could be the main regulators of immune response and exert their functional roles in immune-mediated tissue rejection [31]. Therefore, we used the public microarray datasets to investigate the potential lncRNAs as biomarkers and therapeutic targets for AR after renal transplantation.

In our study, several GEO datasets about AR after renal transplantation were used to identify differentially expressed lncRNAs and mRNAs to construct the DElncRNA-DEmRNA regulatory relationship network based on Pearson correlation analysis. GO and KEGG functional enrichment analyses were performed to reveal the potential biological function of DElncRNAs, showing that T cell activation was mostly enriched. Proliferation and regulation of immune-related cells including leukocytes, lymphocytes, and mononuclear cells were also enriched. These biological processes were achieved probably by the means of interfering cytokine-cytokine receptor interaction pathway and chemokine signaling pathway, which were enriched in KEGG analysis. This suggests that the identified DElncRNAs are deserved to be further explored for the roles in immune response in the future. Then, the DElncRNAs were used for univariate and stepwise multivariate Cox regression analysis and 4 optimal DElncRNAs associated with graft survival were obtained, which included ATP1A1-AS1, EMX2OS, CTD-3080P12.3, and LINC00645. ATP1A1-AS1 is antisense RNA 1 of Na/K-ATPase *α*1 (ATP1A1). In human kidney cells, ATP1A1-AS1 was discovered to negatively regulate its sense gene, ATP1A1, which regulates renal cell survival as a signal transducer [32]. Fan also claimed that ATP1A1-AS1 is a moderate negative regulator of ATP1A1 and modulates Na/K-ATPase-related signaling pathways, which play an important role in cardiac fibrosis [33]. It was also found to be dysregulated expression in cutaneous melanoma [34] and related to the prognosis of thymoma [35]. However, no more studies about the molecular mechanisms and signaling pathways of ATP1A1-AS1 can be found. According to our allograft-infiltrating immune cells analysis, we found that ATP1A1-AS1 is correlated negatively with the infiltration of gamma delta T cells and eosinophils. The role of gamma delta T cells in transplantation is underresearched. Some studies have proven that they frequently present in acute rejection of renal allografts and have direct cytolytic activity against renal epithelium, in which the mechanism for killing allogeneic renal cells is a natural killer-like way [36, 37]. Besides, in small animal models, it was observed that gamma delta T cells could produce interleukin-17 (IL-17) to contribute acute and chronic allograft dysfunction in skin [38] and lung transplantation [39]. In contrast, some evidence showed they could produce IL-4 and IL-10 to decrease Th1 responses to achieve allograft protection [40]. For eosinophils, they are regarded as the promoting factor for inducing acute allograft rejection and the increased presence of eosinophils in peripheral blood and/or renal allograft biopsy specimen would be risky factors for outcome of acute rejection [41, 42]. Therefore, according to what we found, low expression of ATP1A1-AS1 was associated with the high infiltration of gamma delta T cells and eosinophils, which probably provide some hints to reveal the causes for acute rejection of the renal allograft. Besides, our GSEA revealed that ATP1A1-AS1 is involved in allograft rejection and other several signaling pathways including the MAPK signaling pathway [43], MTOR signaling pathway [44], and Toll-like receptor signaling pathway [45], which were discovered to play a role in rejection of renal allograft. EMX2OS is an antisense transcript of homeodomain-containing transcription factor EMX2, which plays a vital role in brain development [46] and urogenital development [47]. Gu et al. revealed the relationship between the downregulation of EMX2OS and poor prognosis of classical papillary thyroid cancer [48], while Tang et al. revealed its participation in the molecular mechanisms regulating recurrent laryngeal cancer [49]. However, there have been little related researches investigating the roles of EMX2OS in kidney cells and even renal allograft with AR. As our study results indicated, downregulation of EMX2OS can predict the poor renal allograft survival after biopsy. It also correlates negatively with gamma delta T cells and eosinophils, which can be risky factors in AR of renal allograft. GSEA suggests that EMX2OS can probably exert its roles through regulation of protein binding, RNA splicing, and transcription factor binding. Besides, low expression of EMX2OS also promotes leukocyte transendothelial migration and the TGF-*β* signaling pathway which regulates cell development, differentiation, apoptosis, and other functions associated with cell homeostasis [50]. As for CTD-3080P12.3, there are not much research introducing its basic information and studying its biological function. Kim et al. regarded CTD-3080P12.3 as a candidate biomarker for thyroid cancer by analyzing their collected samples and The Cancer Genome Atlas dataset [51]. Esposti et al. found it was a differentially expressed lncRNA in hepatocellular carcinoma compared with adjacent cirrhotic tissues [52]. However, the role of CTD-3080P12.3 in rejection or immune response remains unknown. Our results provided some potential clues that CTD-3080P12.3 can be an independent prognostic factor for predicting the renal graft survival as shown in Table 2. The low expression of CTD-3080P12.3 is also associated with the high infiltration of gamma delta T cells and eosinophils in renal allograft with AR. Then on the basis of the functional enrichment analysis, the downregulation of CTD-3080P12.3 possibly gives positively impact on the JAK-STAT signaling pathway, NOD-like receptor signaling pathway, p53 signaling pathway, and Toll-like receptor signaling pathway. The JAK-STAT signaling pathway has the cardinal role in the development and/or function of immune cells [53], while the NOD-like receptor and Toll-like receptor signaling pathways play critical role in activating innate and adaptive immune response [54]. These findings suggest that CTD-3080P12.3 can exert its important role in immune-related aspects. LINC00645, a long intergenic noncoding RNA 645 located in human chromosome 14, is firstly discovered and found to be oncogenic in endometrial cancer by the team of Chen et al. [55]. They found that it was upregulated in endometrial cancer through sequencing the lncRNA transcriptome. Besides, it was studied thoroughly by Li et al., who explored that LINC00645 plays an oncogenic role in glioma through LINC00645/miR-205-3p/ZEB1 signaling axis triggered by the TGF-*β* signaling pathway [56]. However, the action of LINC00645 in renal allograft rejection or immune response remains unclear. Based on our findings, the low expression of LINC00645 is associated with the high infiltration of CD8^+^ T cells and low infiltration of resting dendritic cells. In grafts undergoing acute rejection, invading CD8^+^ T cells with immunologic specificity for the allograft can release perforin and granzymes A and B to perforate target cell membrane and induce caspase-mediated apoptosis of tubular cells [57]. Dendritic cells are critical in the induction of T cell immunity and in peripheral T cell tolerance. It has been reported that resting dendritic cells can induce peripheral CD8^+^ T cells tolerance through PD-1 and CTLA-4 [58, 59]. These discoveries verify what we found for LINC00645, whose low expression will probably whittle the tolerance of CD8^+^ T cells induced by resting dendritic cells and facilitate AR of renal allograft. Besides, our GSEA revealed that “allograft rejection” was enriched by the low expression of LINC00645, while the JAK-STAT signaling pathway and p53 signaling pathway are also enriched, which suggests that it is quite possible to play an important role in AR through these important signaling pathways. From the above, the researches about specific function and mechanism of these four novel lncRNAs remain limited. Our findings provide some important insights for the lncRNAs and more basic researches are recommended to be achieved to investigate their precise role in acute rejection of renal allograft.

After identifying the optimal 4 lncRNAs, logistic regression analysis showed their significant association with acute kidney transplant rejection and their AUC values of ROC analysis interpreted that they have good sensitivity and specificity for differentiating AR of renal allograft (Table 3). Besides, we also constructed a prognosis-related risk score system on the foundation of the 4 lncRNAs to predict the risk of renal graft loss after biopsy. Through calculating the risk score of transplant patients using the formula we provided in the result, the patients in the training set who were classified into the high-risk group were easier to get renal allograft loss than those into the low-risk group. The same significant result was revealed in the testing set. Time-dependent ROC analysis of the risk score system in the training set and the testing set both illustrated the good performance in prediction of 1-, 2-, and 3-year graft survival after biopsy. Therefore, our 4-lncRNA based model may help predict the graft survival outcome of kidney transplant patients and provide the reference for therapeutic guidance in AR of kidney transplant.

However, several limitations should be acknowledged in our studies. Firstly, to the best of our knowledge, there is only one GEO dataset (GSE21374 dataset) which contains survival time of renal graft after biopsy. Thus, in our study, we divided this dataset randomly into the training set and the testing set. The testing set was used to verify the performance of the model screened from the training set. More independent datasets are needed for validation. Secondly, the datasets we used in our study are mainly from North America, which suggests that more public datasets should get involved to eliminate the geographical difference. Thirdly, the optimal biomarkers for AR and graft loss in our study were screened through mining the public dataset merely. The essential clinical parameters supplied by these public datasets are restricted and it is not able to investigate the potential influence of the clinical parameters on the four lncRNAs and the model. Probably in the future we will plan to collect our own data exploring the effects to elaborate the model. According to what we have found, it is still valuable and crucial to conduct the functional experiment in the future to explore the potential mechanism of these ideal four lncRNAs in acute rejection, which probably assists its transition from experimental research to clinical implementation.

## 5. Conclusion

In summary, we used high-throughput microarray datasets to identify 4 novel lncRNAs as biomarkers for AR of renal allograft and construct a lncRNA-based risk score model to predict the risk of graft loss after renal transplantation, which probably aids clinicians in choosing or adjusting therapy of immunosuppression after renal transplantation. Besides, functional enrichment analysis and allograft-infiltrating immune cell analysis revealed the value and importance of the exploring experiment in these 4 novel lncRNAs.

## Figures and Tables

**Table 1:** Clinical characteristics of the GEO datasets.

Characteristics	Merged			GSE50058	GSE76882	GSE21374
	GSE34437	GSE75693	Total			
Graft state						
Acute rejection	17	15	32	43	83	—
Stable	16	30	46	58	99	—
Graft outcome						
Failed	—	—	—	—	—	51
Nonfailed	—	—	—	—	—	231
Total	33	45	78	101	182	282

**Table 2:** Univariate and multivariate Cox regression of 39 DElncRNAs in the training set of GSE21374.

Variable	Univariate			Multivariate		
	HR	95% CI	*p* value	HR	95% CI	*p* value
TRG-AS1	1.951	1.426-2.688	<0.001	—	—	—
LINC00645	0.58	0.443-0.758	<0.001	0.549	0.396-0.761	<0.001
LINC01187	0.461	0.305-0.697	<0.001	—	—	—
TCL6	0.202	0.085-0.480	<0.001	—	—	—
DANCR	0.25	0.116-0.540	<0.001	—	—	—
LINC00982	0.437	0.270-0.707	0.001	—	—	—
CTD-3080P12.3	0.159	0.050-0.504	0.002	0.102	0.021-0.505	0.005
EMX2OS	0.212	0.078-0.576	0.002	0.324	0.091-0.916	0.082
TRAM2-AS1	0.236	0.090-0.620	0.003	—	—	—
LINC00671	0.502	0.309-0.817	0.005	—	—	—
WAC-AS1	3.154	1.414-7.036	0.005	—	—	—
ATP1A1-AS1	0.106	0.021-0.537	0.007	0.14	0.020-0.968	0.046
AC112198.1	0.367	0.174-0.773	0.008	—	—	—
WDFY3-AS2	0.682	0.507-0.916	0.011	—	—	—
LINC00886	0.403	0.183-0.892	0.025	—	—	—
AL022344.5	3.228	1.129-9.231	0.029	—	—	—
RPARP-AS1	0.242	0.066-0.890	0.033	—	—	—
SMIM2-AS1	0.468	0.205-1.071	0.072	—	—	—
RUSCI-AS1	2.547	0.879-7.385	0.085	—	—	—
C12orf77	7.544	0.712-79.89	0.093	—	—	—
ITGB2-AS1	1.338	0.952-1.880	0.094	—	—	—
PCED1B-AS1	2.276	0.866-5.986	0.095	—	—	—
LINC00592	3.047	0.810-11.463	0.099	—	—	—
ADIRF-AS1	0.477	0.197-1.154	0.101	—	—	—
AC005523.2	0.391	0.099-1.533	0.177	—	—	—
ZNF213-AS1	0.416	0.110-1.570	0.196	—	—	—
TRHDE-AS1	0.769	0.479-1.235	0.277	—	—	—
LINC00911	2.155	0.377-12.30	0.387	—	—	—
AC092192.1	1.494	0.534-4.418	0.446	—	—	—
ELOVL2-AS1	2.084	0.316-13.740	0.446	—	—	—
GS1-124K5	2.14	0.247-18.58	0.489	—	—	—
EPB41L4A-AS1	0.651	0.182-2.325	0.508	—	—	—
APTR	0.761	0.221-2.623	0.666	—	—	—
LINC01410	0.601	0.053-6.847	0.682	—	—	—
HMMR-AS1	0.698	0.104-4.654	0.711	—	—	—
LINC00472	0.945	0.639-1.398	0.778	—	—	—
LINC01222	0.776	0.114-5.259	0.795	—	—	—
FLJ37453	0.934	0.145-6.001	0.943	—	—	—
SEPSES-AS1	1.033	0.335-3.179	0.955	—	—	—

HR: hazard ratio; CI: confidence interval.

**Table 3:** Logistic regression analyses and ROC analyses of the 4 optimal lncRNAs.

Predictors	Merged GSE^a^ (*n* = 78)		GSE50058 (*n* = 101)	
	OR	AUC (SE)	OR	AUC (SE)
ATP1A1-AS1	0.032^∗∗∗^	0.863 (0.041)^∗∗∗^	0.123^∗∗∗^	0.780 (0.048)^∗∗∗^
CTD-3080P12.3	0.147^∗∗∗^	0.852 (0.044)^∗∗∗^	0.160^∗∗∗^	0.744 (0.049)^∗∗∗^
EMX20S	0.147^∗∗∗^	0.750 (0.060)^∗∗∗^	0.501^∗∗^	0.666 (0.057)^∗∗^
LINC00645	0.232^∗∗∗^	0.756 (0.059)^∗∗∗^	0.455^∗∗∗^	0.718 (0.052)^∗∗∗^

^a^Merged GSE = GSE34437 + GSE75693. ^∗∗∗^*p* value < 0.001. ^∗∗^*p* value < 0.01. ROC: receiver operating characteristics; OR: odds ratios; AUC: area under the curve; SE: standard error.

## Data Availability

Answer: Yes. Comment: The datasets generated and analyzed during the current study are available in the Gene Expression Omnibus database (GEO, https://www.ncbi.nlm.nih.gov/geo/) and CIBERSORT (https://cibersort.stanford.edu/).

## Abbreviations

AR: Acute rejection
lncRNAs: Long noncoding RNAs
DElncRNAs: Differentially expressed long noncoding RNAs
DEmRNAs: Differentially expressed mRNAs
GEO: Gene Expression Omnibus
KM: Kaplan-Meier
FDR: False discovery rate
FC: Fold change
HR: Hazard ratio
CI: Confidence interval
ROC: Receiver operating characteristic
GSEA: Gene set enrichment analysis
CIBERSORT: Cell type identification by estimating relative subsets of RNA transcripts
GO: Gene Ontology
KEGG: Kyoto Encyclopedia of Genes and Genomes.

## Supplementary Materials

Supplementary 1 Appendix 1: Table S1. Clinical information contained in GSE34437, GSE75693, GSE50058, and GSE76882 datasets.

Supplementary 2 Appendix 2: Table S2. Clinical information contained in GSE21374 dataset.

Supplementary 3 Appendix 3: Figure S1. The normalization of the datasets. (A) The merged dataset including GSE34437 and GSE75693 datasets. (B) GSE50058 dataset. (C) GSE76882 dataset. (D) GSE21374 dataset.

Supplementary 4 Appendix 4: Figure S2. Identification of DEmRNAs. (A) The volcano plot and heat map of top 30 DEmRNAs in the merged dataset. (B) The volcano plot and heat map of top 30 DEmRNAs in GSE50058 dataset. (C) The volcano plot and heat map of top 30 DEmRNAs in GSE76882 dataset.

Supplementary 5 Appendix 5: Figure S3. Kaplan-Meier survival analysis of other 13 prognosis-related DElncRNAs screened after univariate Cox proportional hazards regression analysis. (A) TRG-AS1. (B) LINC00187. (C) LINC00982. (D) LINC00886. (E) LINC00671. (F) RPARP-AS1. (G) DANCR. (H) AC112198.1. (I) WDFY3-AS2. (J) AL022344.5. (K) TCL6. (L) WAC-AS1. (M) TRAM2-AS1.
