# Supplementary figures and images for: Identifying 4 Novel lncRNAs as Potential Biomarkers for Acute Rejection and Graft Loss of Renal Allograft
Source: J Immunol Res. 2020 Nov 28;2020:2415374. doi: 10.1155/2020/2415374 (PMC7739051; doi:10.1155/2020/2415374)

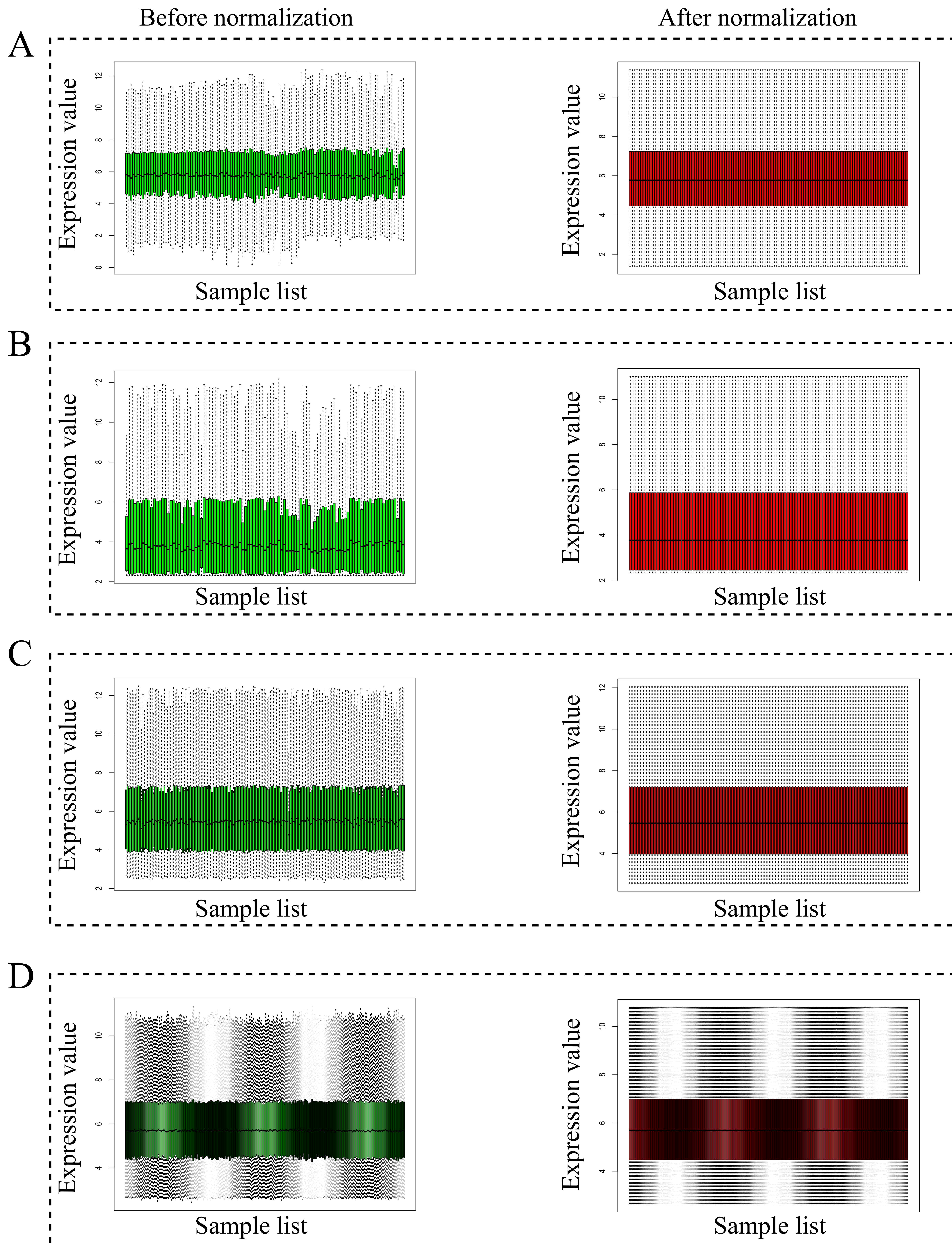

Supplement: Supplementary 1 — Appendix 1: Table S1. Clinical information contained in GSE34437, GSE75693, GSE50058, and GSE76882 datasets. [file 2415374.f1.pdf]

A

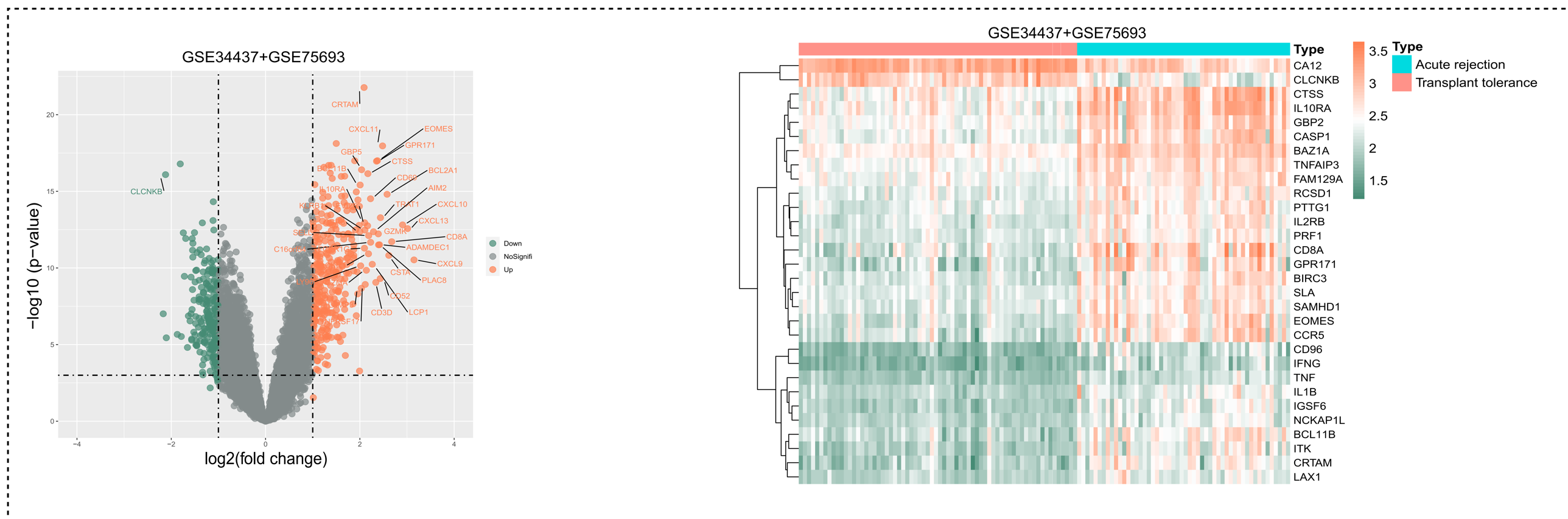

B

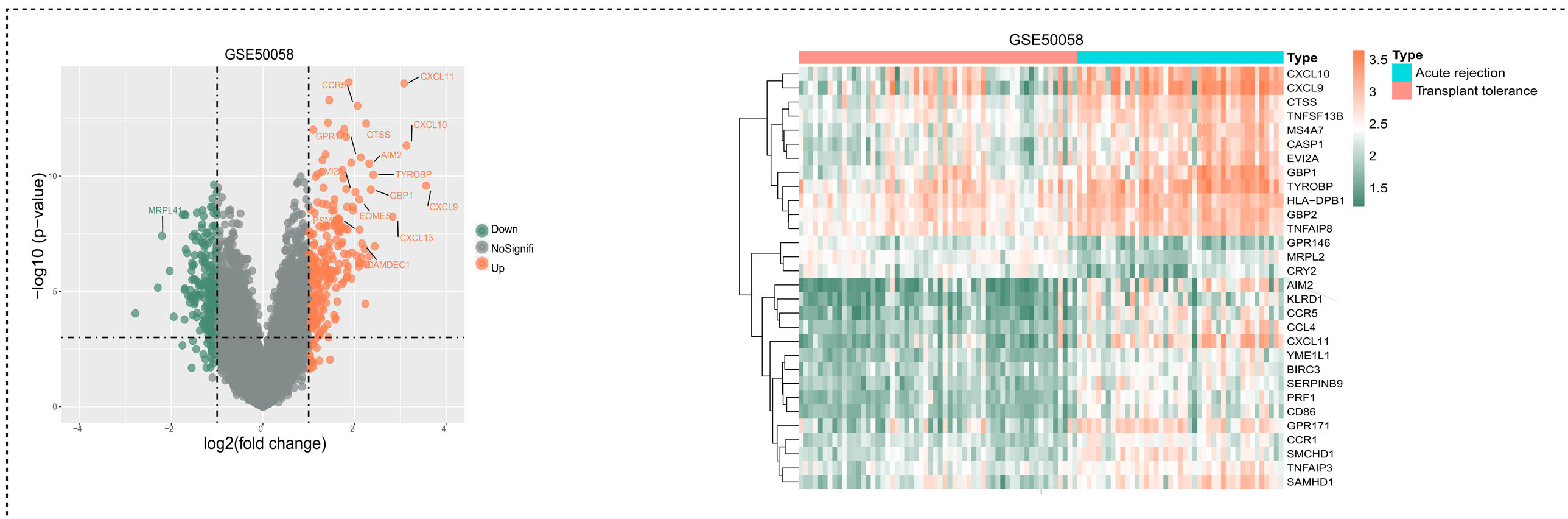

C

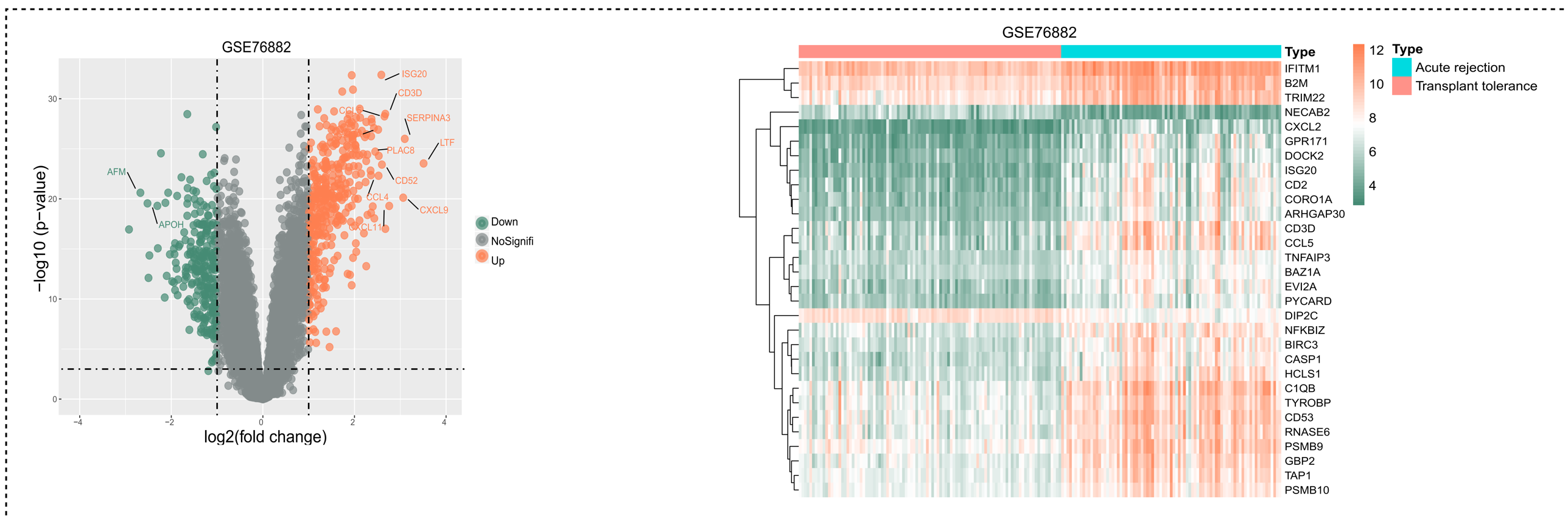

Supplement: Supplementary 2 — Appendix 2: Table S2. Clinical information contained in GSE21374 dataset. [file 2415374.f2.pdf]

A

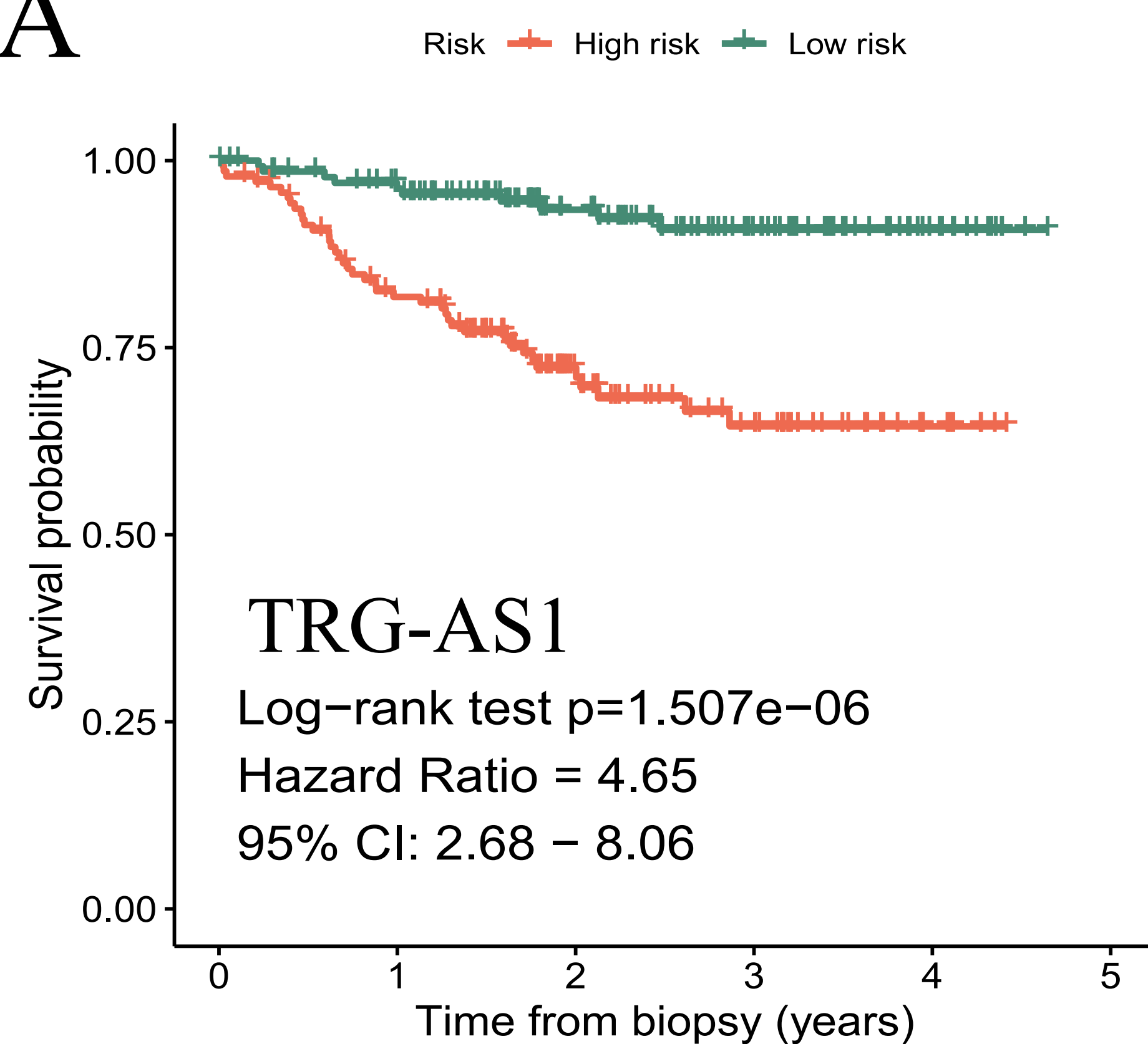

B

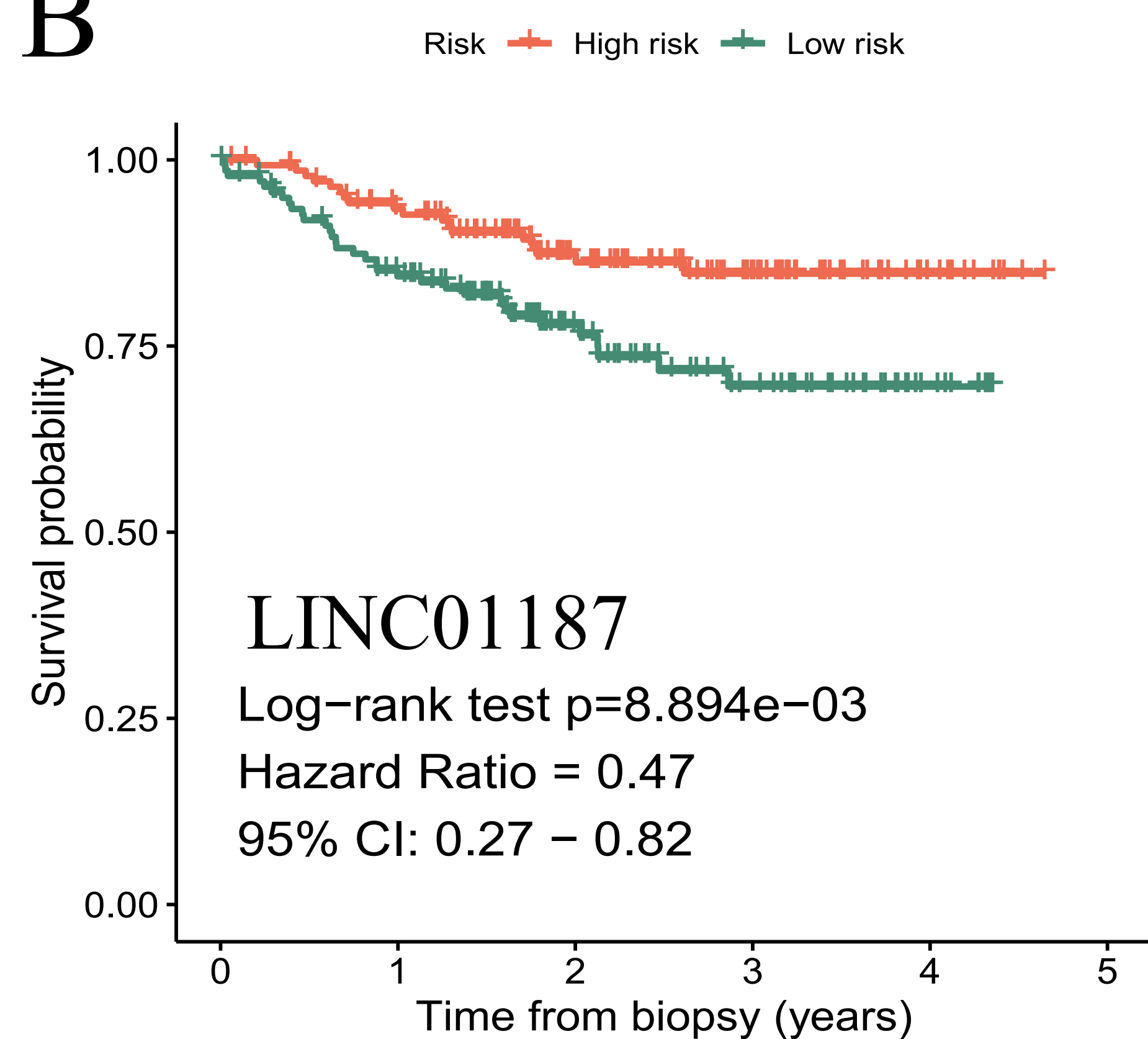

C

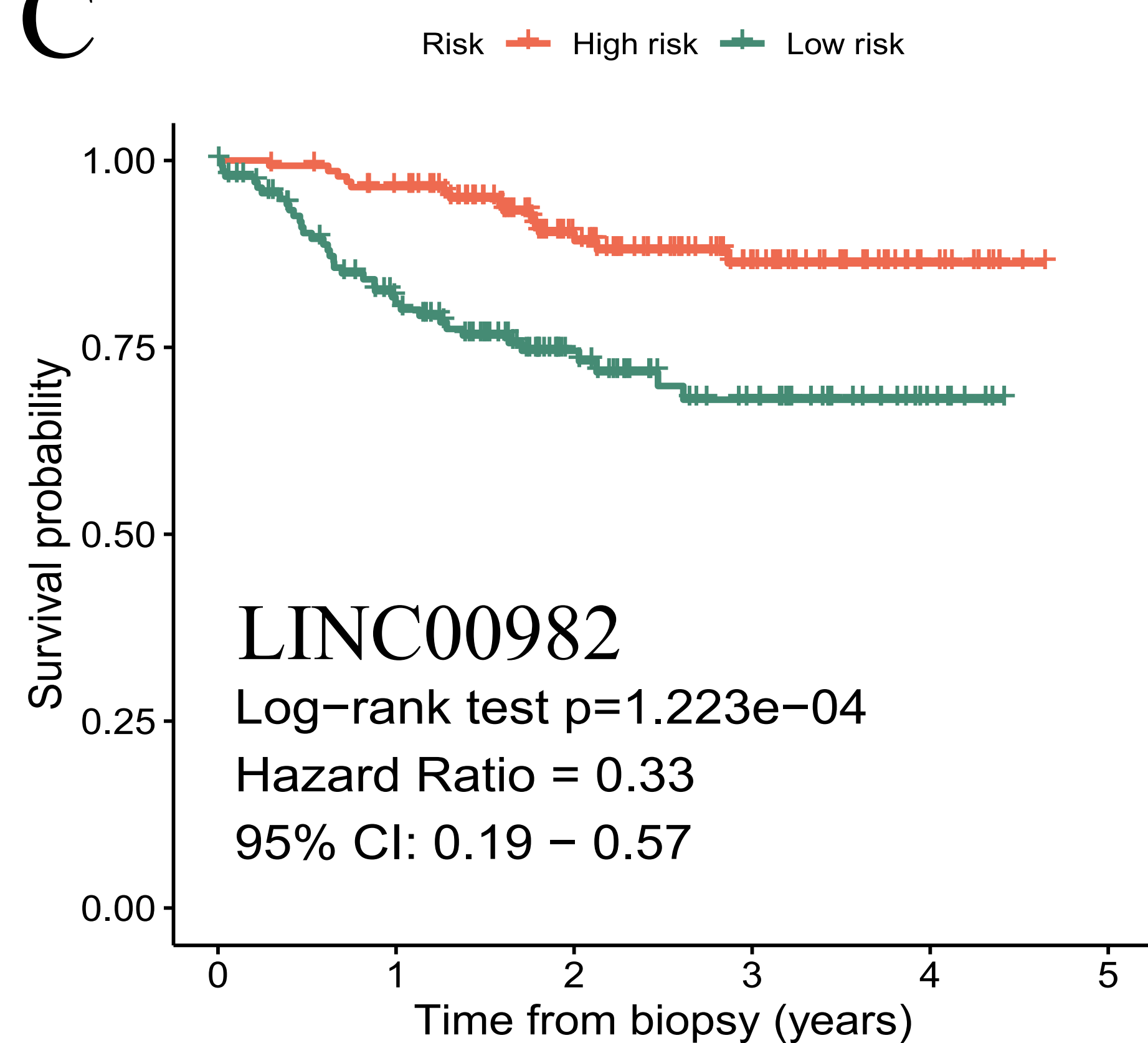

D

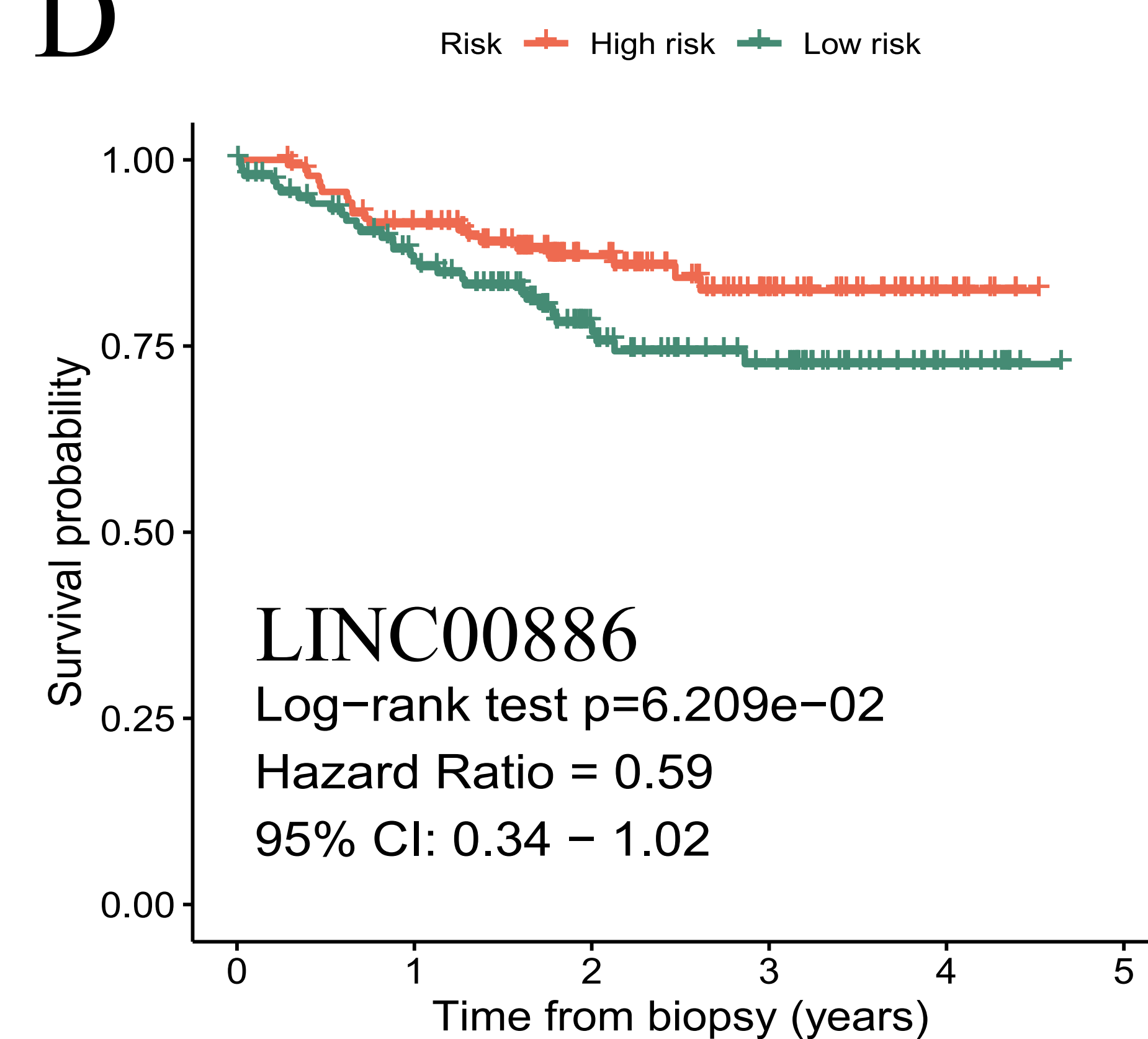

E

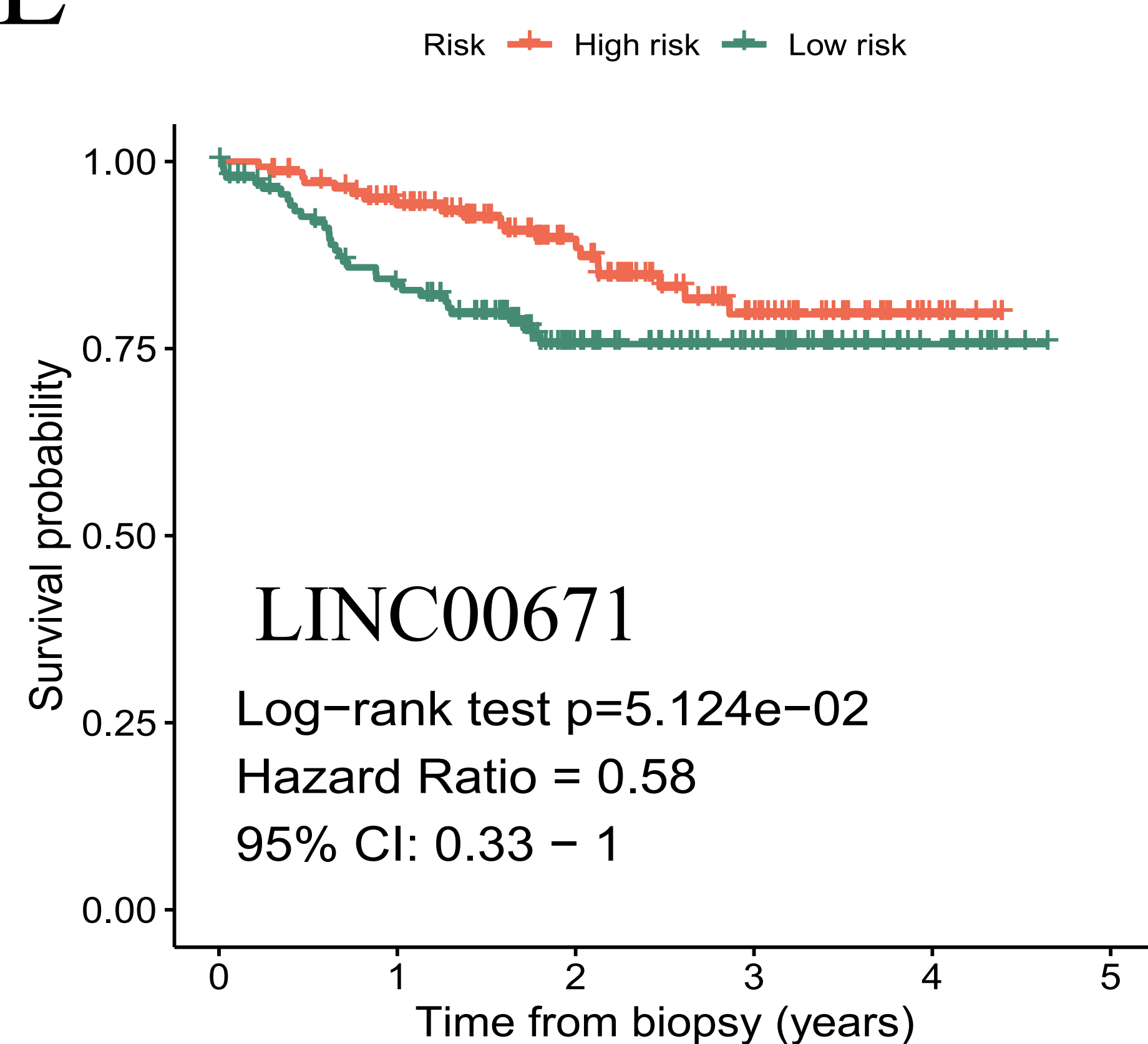

F

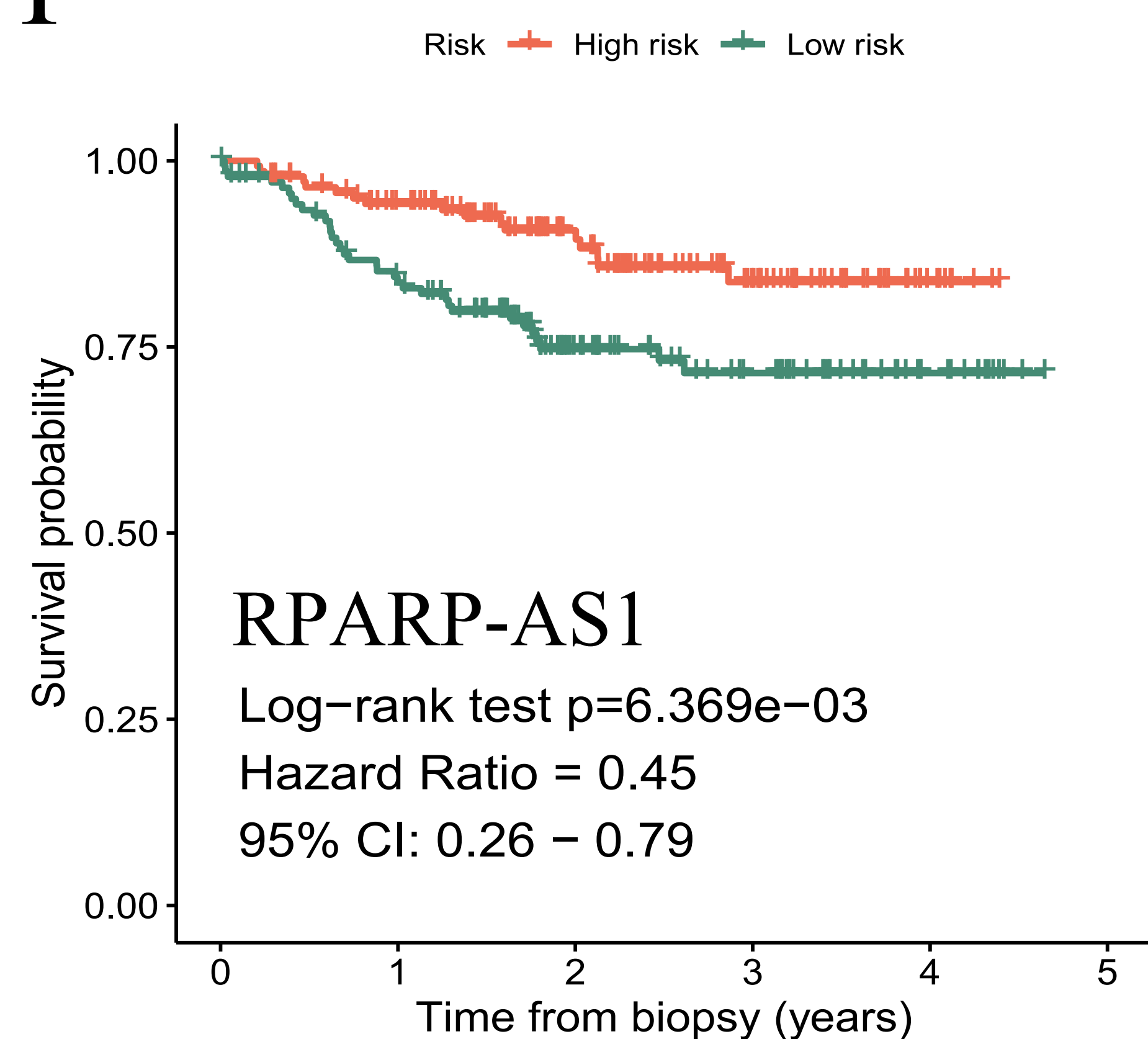

G

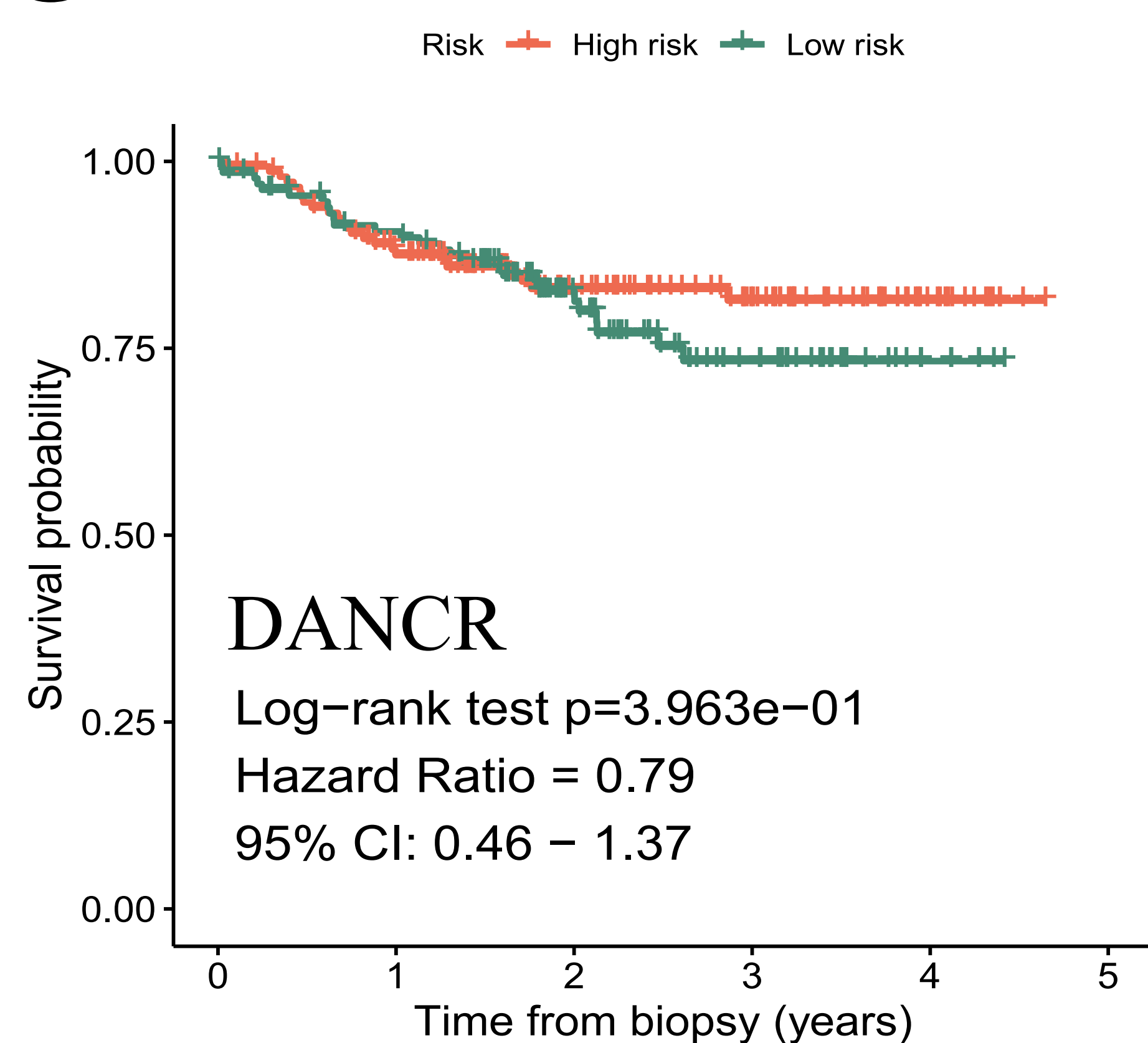

H

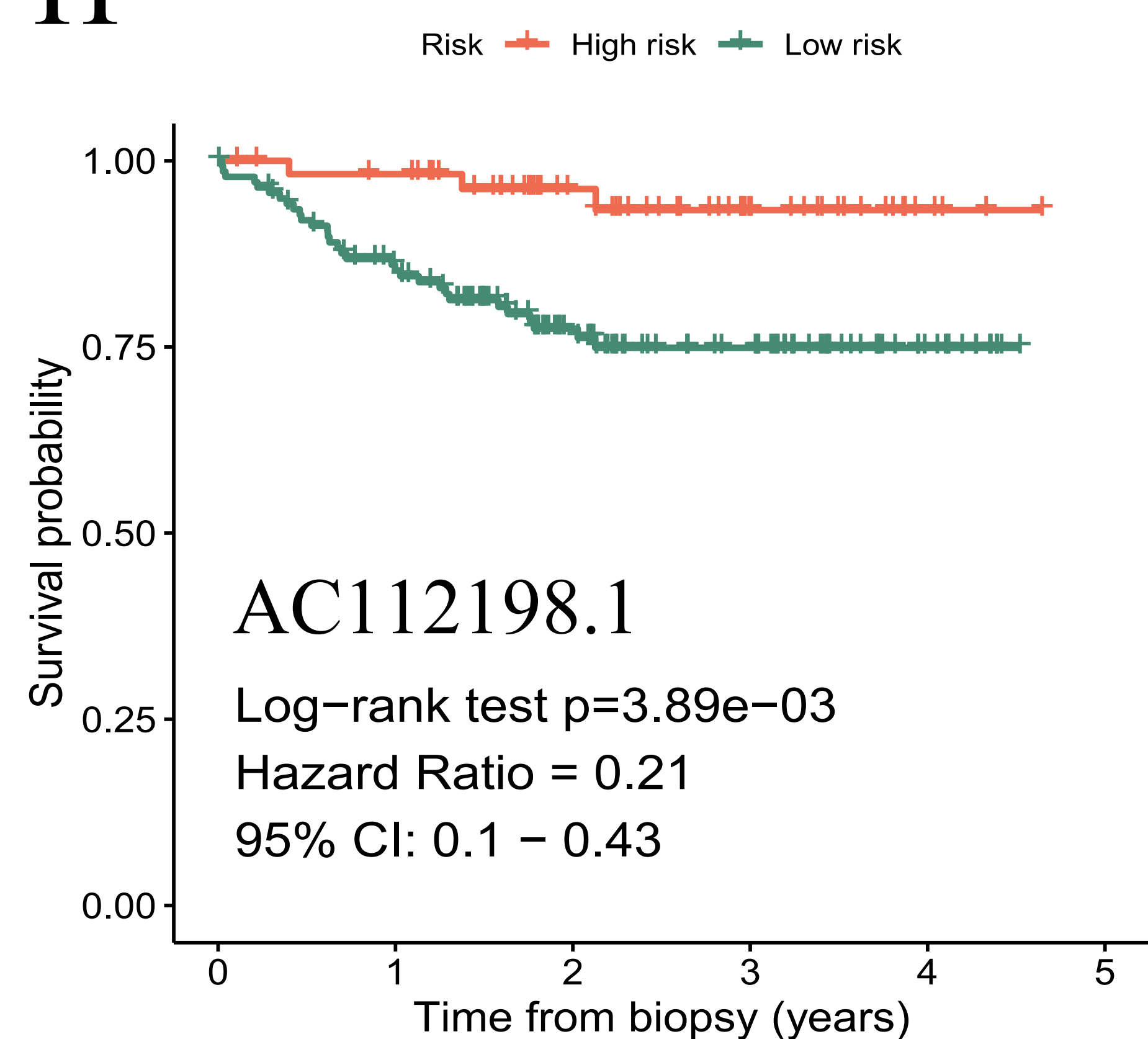

I

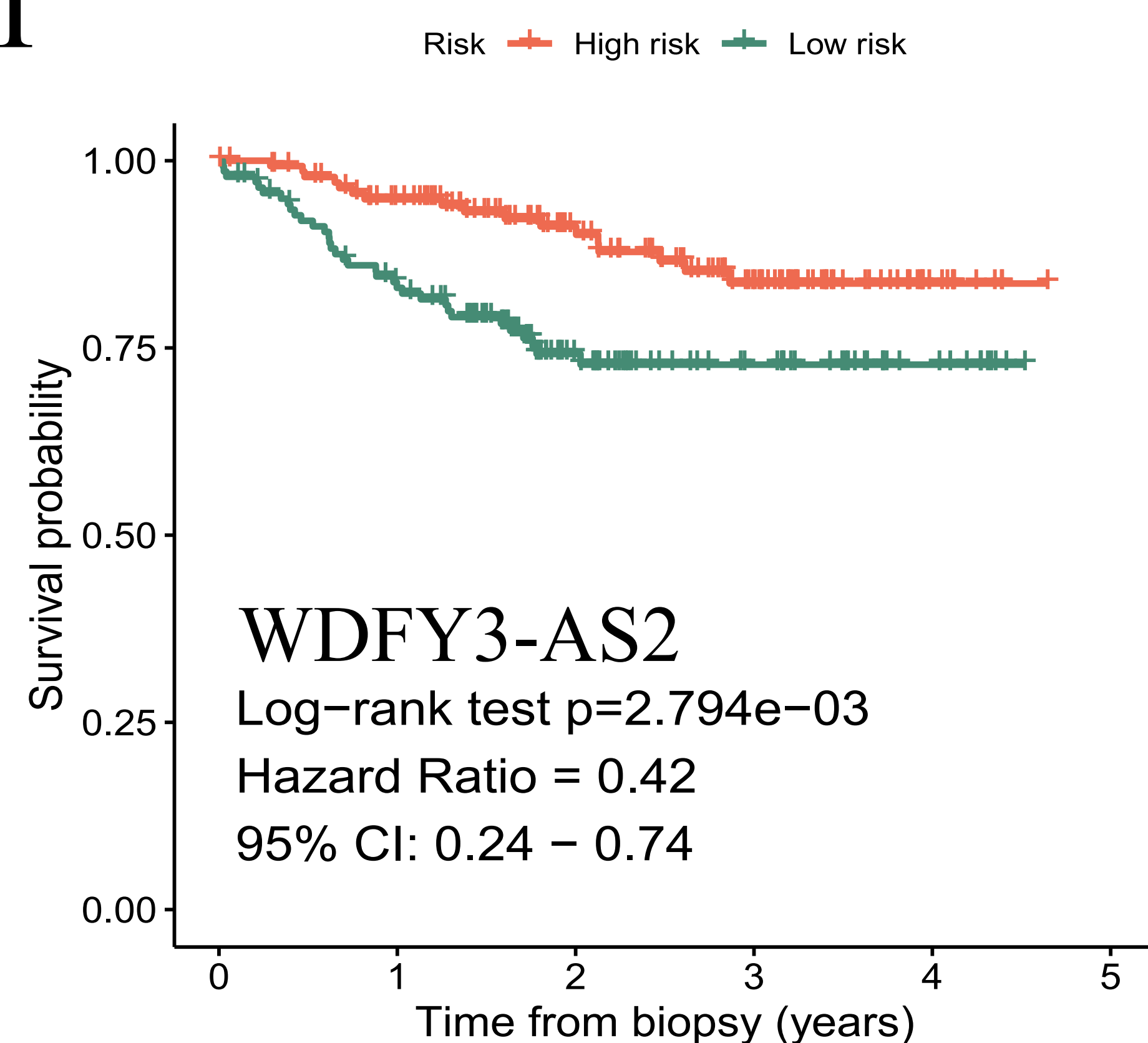

J

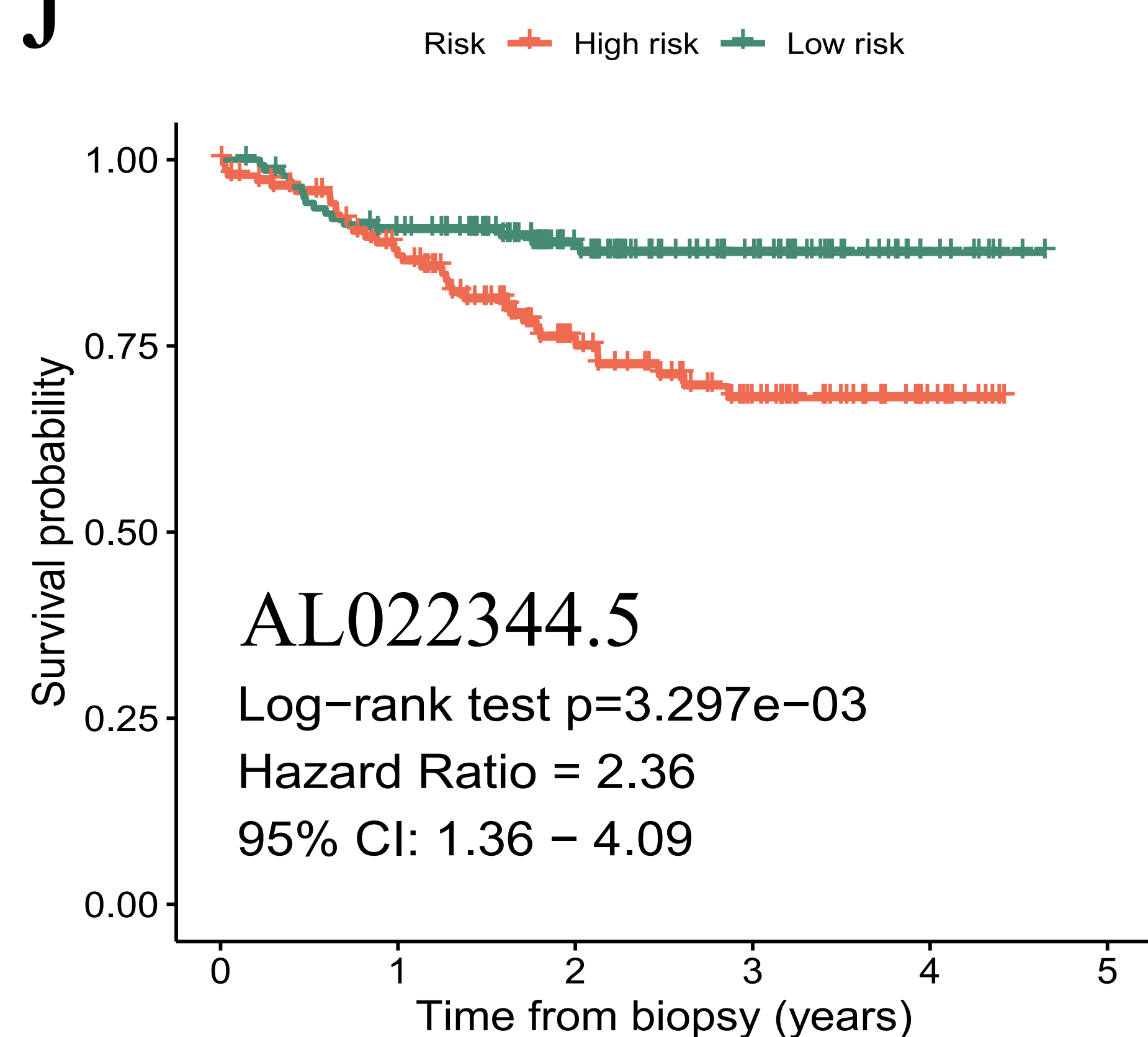

K

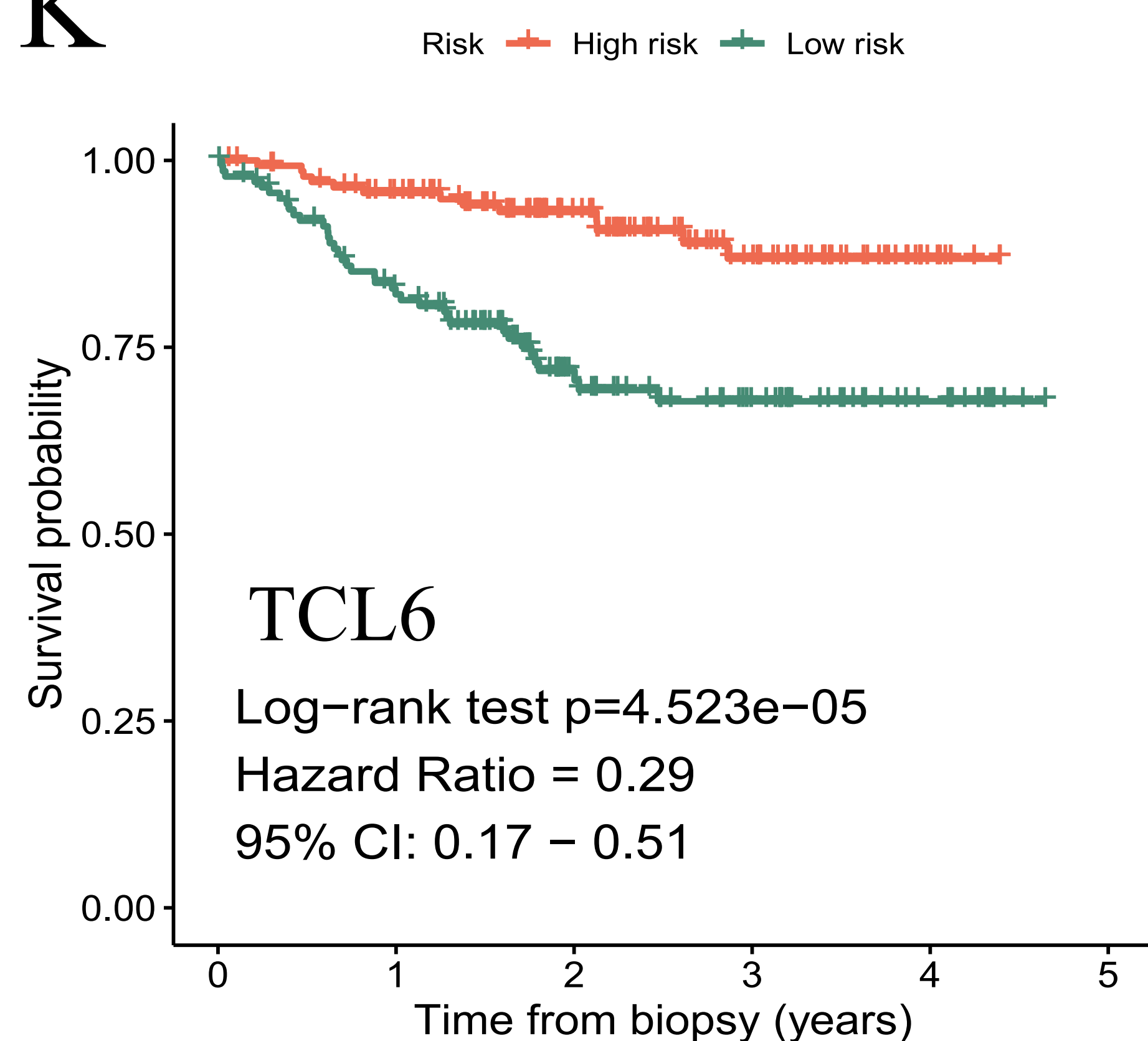

L

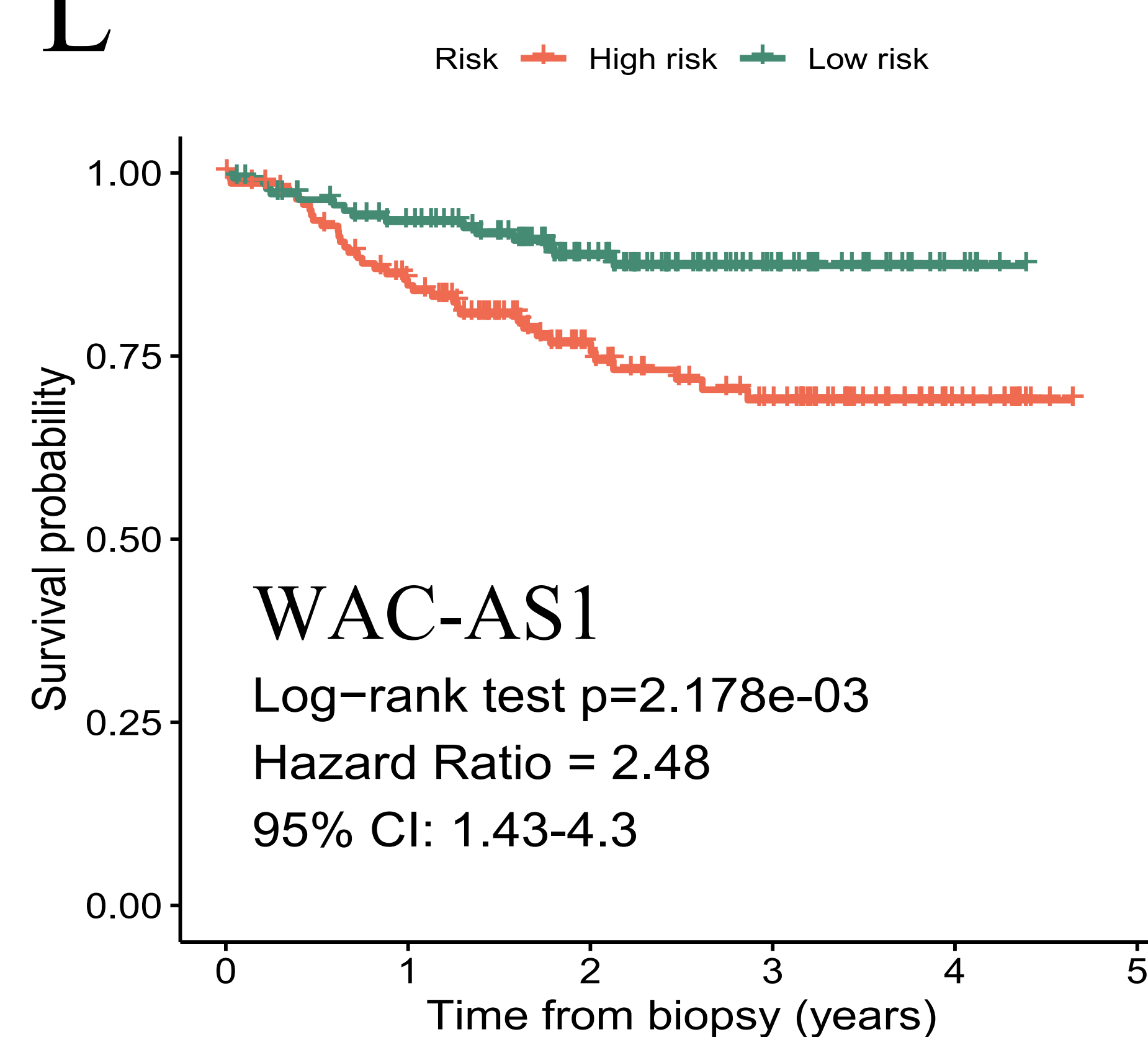

M

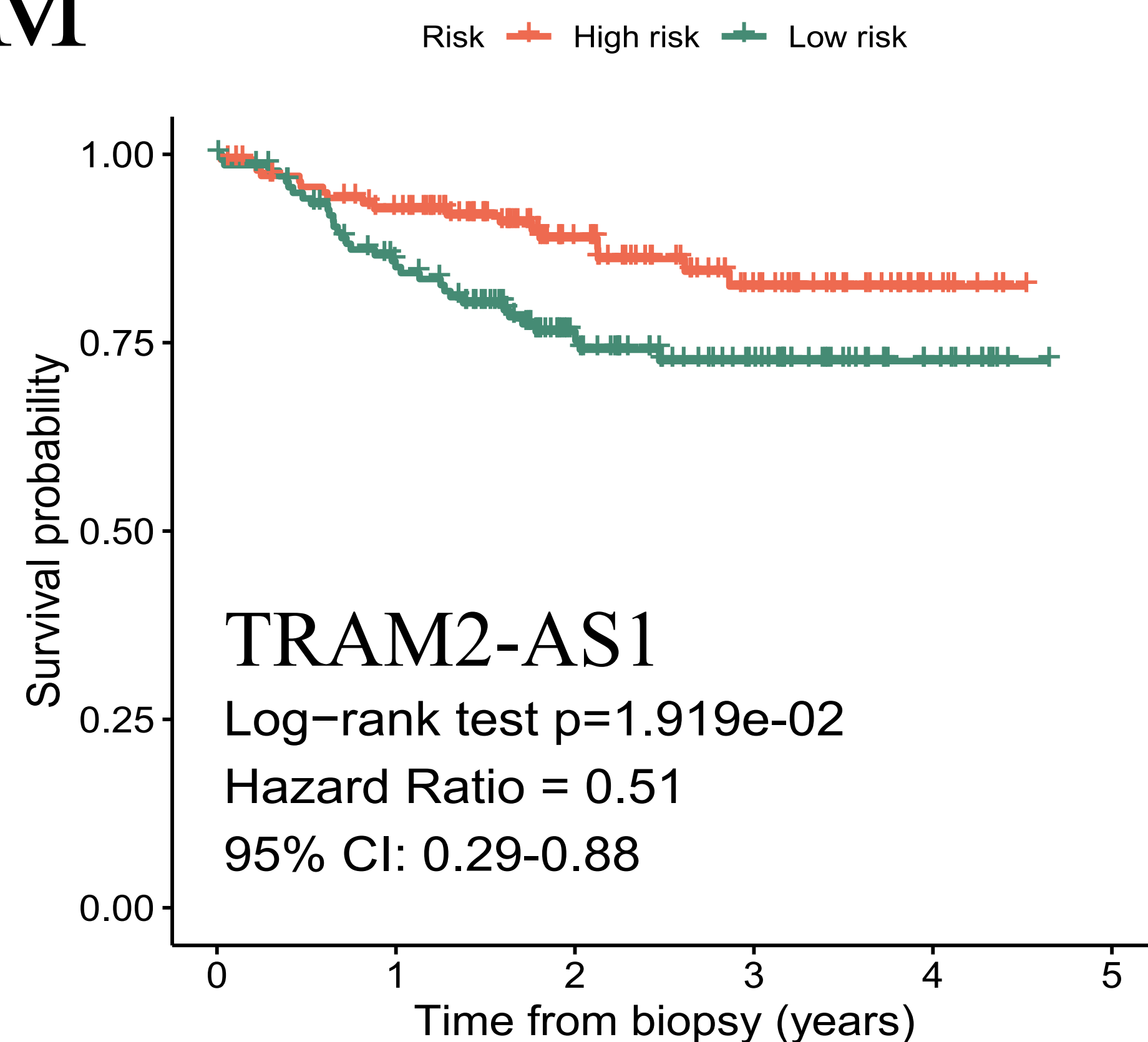

Supplement: Supplementary 3 — Appendix 3: Figure S1. The normalization of the datasets. (A) The merged dataset including GSE34437 and GSE75693 datasets. (B) GSE50058 dataset. (C) GSE76882 dataset. (D) GSE21374 dataset. [file 2415374.f3.pdf]
