# Supplementary material for: Identifying 4 Novel lncRNAs as Potential Biomarkers for Acute Rejection and Graft Loss of Renal Allograft
Source: J Immunol Res. 2020 Nov 28;2020:2415374. doi: 10.1155/2020/2415374 (PMC7739051; doi:10.1155/2020/2415374)
Supplement: Supplementary 4 — Appendix 4: Figure S2. Identification of DEmRNAs. (A) The volcano plot and heat map of top 30 DEmRNAs in the merged dataset. (B) The volcano plot and heat map of top 30 DEmRNAs in GSE50058 dataset. (C) The volcano plot and heat map of top 30 DEmRNAs in GSE76882 dataset. [file 2415374.f4.docx]

| Table S1. Clinical information contained in GSE34437, GSE75693, GSE50058 and GSE76882 datasets. | | | | |
| --- | --- | --- | --- | --- |
| **Parameters** | **GSE34437** | **GSE75693** | **GSE50058** | **GSE76882** |
| **Source** | Allograft biopsy | Allograft biopsy | Allograft biopsy | Allograft biopsy |
| **Organism** | Homo sapiens | Homo sapiens | Homo sapiens | Homo sapiens |
| **Type** | RNA | RNA | RNA | RNA |
| **Tissue** | Kidney | Kidney | Kidney | Kidney |
| **Graft source** | Living donor | Unknown | Living donor | Unknown |
| **Graft state** | No significant abnormalities | Stable graft | Non-rejection | TX |
|  | Baseline, living donor | Absence of chronic allograft nephropathy | AR | ADNR |
|  | Borderline AR | Presence of chronic allograft nephropathy |  | IFTA |
|  | AR | BKVN biopsy |  | IFTA_i |
|  |  | AR |  | AR |
|  |  |  |  | IFTA_AR |
| AR: acute rejection | |  |  |  |
| TX: normal functioning transplants | | |  |  |
| ADNR: acute dysfunction but no rejection | | |  |  |
| IFTA: Interstitial fibrosis and tubular atrophy | | |  |  |
| IFTA_i: Interstitial fibrosis and tubular atrophy with inflammation | | | |  |
| IFTA_AR: Interstitial fibrosis and tubular atrophy with inflammation with acute rejection | | | | |
