# Supplementary material for: Identifying 4 Novel lncRNAs as Potential Biomarkers for Acute Rejection and Graft Loss of Renal Allograft
Source: J Immunol Res. 2020 Nov 28;2020:2415374. doi: 10.1155/2020/2415374 (PMC7739051; doi:10.1155/2020/2415374)
Supplement: Supplementary 5 — Appendix 5: Figure S3. Kaplan-Meier survival analysis of other 13 prognosis-related DElncRNAs screened after univariate Cox proportional hazards regression analysis. (A) TRG-AS1. (B) LINC00187. (C) LINC00982. (D) LINC00886. (E) LINC00671. (F) RPARP-AS1. (G) DANCR. (H) AC112198.1. (I) WDFY3-AS2. (J) AL022344.5. (K) TCL6. (L) WAC-AS1. (M) TRAM2-AS1. [file 2415374.f5.docx]

| Table S2. Clinical information contained in GSE21374 dataset. | | | | | | |  |
| --- | --- | --- | --- | --- | --- | --- | --- |
| **ID** | **Source** | **Organism** | **Molecular** | **Time from biopsy to failure/censoring (Days)** | **Time of biopsy post transplant (Days)** | **Rejection/Non-rejection** | **Graft outcome** |
| GSM533921 | renal allograft biopsy | Homo sapiens | total RNA | 1084 | 277 | nonrejection | unfailed |
| GSM533922 | renal allograft biopsy | Homo sapiens | total RNA | 1170 | 134 | nonrejection | unfailed |
| GSM533923 | renal allograft biopsy | Homo sapiens | total RNA | 127 | 795 | nonrejection | failed |
| GSM533924 | renal allograft biopsy | Homo sapiens | total RNA | 1430 | 2358 | nonrejection | unfailed |
| GSM533925 | renal allograft biopsy | Homo sapiens | total RNA | 1244 | 64 | nonrejection | unfailed |
| GSM533926 | renal allograft biopsy | Homo sapiens | total RNA | 1154 | 8 | rejection | unfailed |
| GSM533927 | renal allograft biopsy | Homo sapiens | total RNA | 1410 | 745 | rejection | unfailed |
| GSM533928 | renal allograft biopsy | Homo sapiens | total RNA | 1254 | 1309 | nonrejection | unfailed |
| GSM533929 | renal allograft biopsy | Homo sapiens | total RNA | 1277 | 1461 | nonrejection | unfailed |
| GSM533930 | renal allograft biopsy | Homo sapiens | total RNA | 652 | 2086 | nonrejection | unfailed |
| GSM533931 | renal allograft biopsy | Homo sapiens | total RNA | 1240 | 3442 | rejection | unfailed |
| GSM533932 | renal allograft biopsy | Homo sapiens | total RNA | 79 | 1591 | nonrejection | unfailed |
| GSM533933 | renal allograft biopsy | Homo sapiens | total RNA | 1276 | 881 | nonrejection | unfailed |
| GSM533934 | renal allograft biopsy | Homo sapiens | total RNA | 1112 | 7 | nonrejection | unfailed |
| GSM533935 | renal allograft biopsy | Homo sapiens | total RNA | 1153 | 117 | rejection | unfailed |
| GSM533936 | renal allograft biopsy | Homo sapiens | total RNA | 659 | 611 | rejection | unfailed |
| GSM533937 | renal allograft biopsy | Homo sapiens | total RNA | 655 | 615 | rejection | unfailed |
| GSM533938 | renal allograft biopsy | Homo sapiens | total RNA | 1258 | 1703 | nonrejection | unfailed |
| GSM533939 | renal allograft biopsy | Homo sapiens | total RNA | 1167 | 42 | nonrejection | unfailed |
| GSM533940 | renal allograft biopsy | Homo sapiens | total RNA | 928 | 281 | nonrejection | unfailed |
| GSM533941 | renal allograft biopsy | Homo sapiens | total RNA | 1138 | 36 | nonrejection | unfailed |
| GSM533942 | renal allograft biopsy | Homo sapiens | total RNA | 907 | 178 | nonrejection | unfailed |
| GSM533943 | renal allograft biopsy | Homo sapiens | total RNA | 1234 | 85 | nonrejection | unfailed |
| GSM533944 | renal allograft biopsy | Homo sapiens | total RNA | 1176 | 7387 | nonrejection | unfailed |
| GSM533945 | renal allograft biopsy | Homo sapiens | total RNA | 1143 | 398 | nonrejection | unfailed |
| GSM533946 | renal allograft biopsy | Homo sapiens | total RNA | 1185 | 2094 | nonrejection | unfailed |
| GSM533947 | renal allograft biopsy | Homo sapiens | total RNA | 1182 | 1242 | nonrejection | unfailed |
| GSM533948 | renal allograft biopsy | Homo sapiens | total RNA | 1181 | 2502 | nonrejection | unfailed |
| GSM533949 | renal allograft biopsy | Homo sapiens | total RNA | 1051 | 4120 | nonrejection | unfailed |
| GSM533950 | renal allograft biopsy | Homo sapiens | total RNA | 1206 | 2533 | nonrejection | unfailed |
| GSM533951 | renal allograft biopsy | Homo sapiens | total RNA | 585 | 2404 | rejection | failed |
| GSM533952 | renal allograft biopsy | Homo sapiens | total RNA | 237 | 2752 | rejection | failed |
| GSM533953 | renal allograft biopsy | Homo sapiens | total RNA | 1171 | 1524 | nonrejection | unfailed |
| GSM533954 | renal allograft biopsy | Homo sapiens | total RNA | 1035 | 3584 | nonrejection | unfailed |
| GSM533955 | renal allograft biopsy | Homo sapiens | total RNA | 740 | 603 | rejection | failed |
| GSM533956 | renal allograft biopsy | Homo sapiens | total RNA | 658 | 685 | rejection | failed |
| GSM533957 | renal allograft biopsy | Homo sapiens | total RNA | 775 | 240 | rejection | unfailed |
| GSM533958 | renal allograft biopsy | Homo sapiens | total RNA | 1148 | 3204 | nonrejection | unfailed |
| GSM533959 | renal allograft biopsy | Homo sapiens | total RNA | 1002 | 4445 | rejection | unfailed |
| GSM533960 | renal allograft biopsy | Homo sapiens | total RNA | 1110 | 110 | nonrejection | unfailed |
| GSM533961 | renal allograft biopsy | Homo sapiens | total RNA | 1036 | 79 | nonrejection | unfailed |
| GSM533962 | renal allograft biopsy | Homo sapiens | total RNA | 1124 | 73 | nonrejection | unfailed |
| GSM533963 | renal allograft biopsy | Homo sapiens | total RNA | 364 | 3775 | rejection | failed |
| GSM533964 | renal allograft biopsy | Homo sapiens | total RNA | 456 | 742 | rejection | failed |
| GSM533965 | renal allograft biopsy | Homo sapiens | total RNA | 1022 | 100 | nonrejection | unfailed |
| GSM533966 | renal allograft biopsy | Homo sapiens | total RNA | 777 | 345 | nonrejection | unfailed |
| GSM533967 | renal allograft biopsy | Homo sapiens | total RNA | 731 | 2384 | nonrejection | failed |
| GSM533968 | renal allograft biopsy | Homo sapiens | total RNA | 1106 | 630 | nonrejection | unfailed |
| GSM533969 | renal allograft biopsy | Homo sapiens | total RNA | 1590 | 26 | nonrejection | unfailed |
| GSM533970 | renal allograft biopsy | Homo sapiens | total RNA | 827 | 287 | nonrejection | unfailed |
| GSM533971 | renal allograft biopsy | Homo sapiens | total RNA | 727 | 387 | nonrejection | unfailed |
| GSM533972 | renal allograft biopsy | Homo sapiens | total RNA | 712 | 402 | nonrejection | unfailed |
| GSM533973 | renal allograft biopsy | Homo sapiens | total RNA | 902 | 2559 | nonrejection | failed |
| GSM533974 | renal allograft biopsy | Homo sapiens | total RNA | 1078 | 3617 | nonrejection | unfailed |
| GSM533975 | renal allograft biopsy | Homo sapiens | total RNA | 507 | 553 | rejection | unfailed |
| GSM533976 | renal allograft biopsy | Homo sapiens | total RNA | 1002 | 162 | rejection | unfailed |
| GSM533977 | renal allograft biopsy | Homo sapiens | total RNA | 1156 | 8 | rejection | unfailed |
| GSM533978 | renal allograft biopsy | Homo sapiens | total RNA | 1093 | 71 | rejection | unfailed |
| GSM533979 | renal allograft biopsy | Homo sapiens | total RNA | 1030 | 134 | nonrejection | unfailed |
| GSM533980 | renal allograft biopsy | Homo sapiens | total RNA | 1216 | 1148 | rejection | unfailed |
| GSM533981 | renal allograft biopsy | Homo sapiens | total RNA | 702 | 1662 | nonrejection | unfailed |
| GSM533982 | renal allograft biopsy | Homo sapiens | total RNA | 1323 | 1041 | nonrejection | unfailed |
| GSM533983 | renal allograft biopsy | Homo sapiens | total RNA | 887 | 4726 | nonrejection | unfailed |
| GSM533984 | renal allograft biopsy | Homo sapiens | total RNA | 981 | 4135 | nonrejection | unfailed |
| GSM533985 | renal allograft biopsy | Homo sapiens | total RNA | 873 | 4811 | nonrejection | unfailed |
| GSM533986 | renal allograft biopsy | Homo sapiens | total RNA | 1011 | 46 | nonrejection | unfailed |
| GSM533987 | renal allograft biopsy | Homo sapiens | total RNA | 979 | 78 | nonrejection | unfailed |
| GSM533988 | renal allograft biopsy | Homo sapiens | total RNA | 546 | 511 | rejection | unfailed |
| GSM533989 | renal allograft biopsy | Homo sapiens | total RNA | 523 | 534 | rejection | unfailed |
| GSM533990 | renal allograft biopsy | Homo sapiens | total RNA | 1440 | 216 | rejection | unfailed |
| GSM533991 | renal allograft biopsy | Homo sapiens | total RNA | 1414 | 242 | nonrejection | unfailed |
| GSM533992 | renal allograft biopsy | Homo sapiens | total RNA | 951 | 3361 | nonrejection | unfailed |
| GSM533993 | renal allograft biopsy | Homo sapiens | total RNA | 254 | 6468 | nonrejection | failed |
| GSM533994 | renal allograft biopsy | Homo sapiens | total RNA | 175 | 6547 | nonrejection | failed |
| GSM533995 | renal allograft biopsy | Homo sapiens | total RNA | 246 | 678 | rejection | failed |
| GSM533996 | renal allograft biopsy | Homo sapiens | total RNA | 945 | 14 | nonrejection | unfailed |
| GSM533997 | renal allograft biopsy | Homo sapiens | total RNA | 1076 | 743 | nonrejection | unfailed |
| GSM533998 | renal allograft biopsy | Homo sapiens | total RNA | 882 | 922 | nonrejection | unfailed |
| GSM533999 | renal allograft biopsy | Homo sapiens | total RNA | 905 | 85 | nonrejection | unfailed |
| GSM534000 | renal allograft biopsy | Homo sapiens | total RNA | 746 | 57 | nonrejection | unfailed |
| GSM534001 | renal allograft biopsy | Homo sapiens | total RNA | 590 | 161 | rejection | unfailed |
| GSM534002 | renal allograft biopsy | Homo sapiens | total RNA | 936 | 5744 | nonrejection | unfailed |
| GSM534003 | renal allograft biopsy | Homo sapiens | total RNA | 967 | 3082 | nonrejection | unfailed |
| GSM534004 | renal allograft biopsy | Homo sapiens | total RNA | 884 | 46 | nonrejection | unfailed |
| GSM534005 | renal allograft biopsy | Homo sapiens | total RNA | 464 | 3878 | rejection | failed |
| GSM534006 | renal allograft biopsy | Homo sapiens | total RNA | 10 | 6938 | nonrejection | failed |
| GSM534007 | renal allograft biopsy | Homo sapiens | total RNA | 463 | 306 | rejection | unfailed |
| GSM534008 | renal allograft biopsy | Homo sapiens | total RNA | 322 | 465 | rejection | failed |
| GSM534009 | renal allograft biopsy | Homo sapiens | total RNA | 273 | 514 | rejection | failed |
| GSM534010 | renal allograft biopsy | Homo sapiens | total RNA | 901 | 5985 | rejection | unfailed |
| GSM534011 | renal allograft biopsy | Homo sapiens | total RNA | 820 | 846 | nonrejection | unfailed |
| GSM534012 | renal allograft biopsy | Homo sapiens | total RNA | 453 | 1213 | nonrejection | unfailed |
| GSM534013 | renal allograft biopsy | Homo sapiens | total RNA | 1696 | 8 | nonrejection | unfailed |
| GSM534014 | renal allograft biopsy | Homo sapiens | total RNA | 630 | 245 | rejection | unfailed |
| GSM534015 | renal allograft biopsy | Homo sapiens | total RNA | 881 | 969 | nonrejection | unfailed |
| GSM534016 | renal allograft biopsy | Homo sapiens | total RNA | 837 | 2650 | rejection | unfailed |
| GSM534017 | renal allograft biopsy | Homo sapiens | total RNA | 811 | 2676 | nonrejection | unfailed |
| GSM534018 | renal allograft biopsy | Homo sapiens | total RNA | 650 | 5414 | nonrejection | failed |
| GSM534019 | renal allograft biopsy | Homo sapiens | total RNA | 698 | 23 | nonrejection | unfailed |
| GSM534020 | renal allograft biopsy | Homo sapiens | total RNA | 719 | 2022 | rejection | unfailed |
| GSM534021 | renal allograft biopsy | Homo sapiens | total RNA | 698 | 2043 | nonrejection | unfailed |
| GSM534022 | renal allograft biopsy | Homo sapiens | total RNA | 768 | 66 | nonrejection | unfailed |
| GSM534023 | renal allograft biopsy | Homo sapiens | total RNA | 741 | 93 | rejection | unfailed |
| GSM534024 | renal allograft biopsy | Homo sapiens | total RNA | 708 | 126 | rejection | unfailed |
| GSM534025 | renal allograft biopsy | Homo sapiens | total RNA | 652 | 182 | nonrejection | unfailed |
| GSM534026 | renal allograft biopsy | Homo sapiens | total RNA | 469 | 3760 | nonrejection | failed |
| GSM534027 | renal allograft biopsy | Homo sapiens | total RNA | 844 | 1093 | nonrejection | unfailed |
| GSM534028 | renal allograft biopsy | Homo sapiens | total RNA | 581 | 544 | rejection | unfailed |
| GSM534029 | renal allograft biopsy | Homo sapiens | total RNA | 1650 | 183 | nonrejection | unfailed |
| GSM534030 | renal allograft biopsy | Homo sapiens | total RNA | 1603 | 230 | nonrejection | unfailed |
| GSM534031 | renal allograft biopsy | Homo sapiens | total RNA | 1503 | 330 | nonrejection | unfailed |
| GSM534032 | renal allograft biopsy | Homo sapiens | total RNA | 697 | 148 | rejection | unfailed |
| GSM534033 | renal allograft biopsy | Homo sapiens | total RNA | 670 | 175 | rejection | unfailed |
| GSM534034 | renal allograft biopsy | Homo sapiens | total RNA | 375 | 7730 | nonrejection | failed |
| GSM534035 | renal allograft biopsy | Homo sapiens | total RNA | 762 | 58 | nonrejection | unfailed |
| GSM534036 | renal allograft biopsy | Homo sapiens | total RNA | 75 | 742 | nonrejection | failed |
| GSM534037 | renal allograft biopsy | Homo sapiens | total RNA | 575 | 8973 | nonrejection | unfailed |
| GSM534038 | renal allograft biopsy | Homo sapiens | total RNA | 700 | 302 | nonrejection | unfailed |
| GSM534039 | renal allograft biopsy | Homo sapiens | total RNA | 1559 | 1118 | rejection | unfailed |
| GSM534040 | renal allograft biopsy | Homo sapiens | total RNA | 144 | 2526 | nonrejection | unfailed |
| GSM534041 | renal allograft biopsy | Homo sapiens | total RNA | 693 | 13 | nonrejection | unfailed |
| GSM534042 | renal allograft biopsy | Homo sapiens | total RNA | 694 | 2847 | nonrejection | unfailed |
| GSM534043 | renal allograft biopsy | Homo sapiens | total RNA | 413 | 541 | rejection | failed |
| GSM534044 | renal allograft biopsy | Homo sapiens | total RNA | 142 | 633 | nonrejection | unfailed |
| GSM534045 | renal allograft biopsy | Homo sapiens | total RNA | 579 | 654 | nonrejection | unfailed |
| GSM534046 | renal allograft biopsy | Homo sapiens | total RNA | 508 | 725 | nonrejection | unfailed |
| GSM534047 | renal allograft biopsy | Homo sapiens | total RNA | 773 | 21 | nonrejection | unfailed |
| GSM534048 | renal allograft biopsy | Homo sapiens | total RNA | 766 | 28 | nonrejection | unfailed |
| GSM534049 | renal allograft biopsy | Homo sapiens | total RNA | 659 | 135 | nonrejection | unfailed |
| GSM534050 | renal allograft biopsy | Homo sapiens | total RNA | 141 | 1700 | nonrejection | failed |
| GSM534051 | renal allograft biopsy | Homo sapiens | total RNA | 817 | 6 | nonrejection | unfailed |
| GSM534052 | renal allograft biopsy | Homo sapiens | total RNA | 680 | 278 | nonrejection | unfailed |
| GSM534053 | renal allograft biopsy | Homo sapiens | total RNA | 639 | 12831 | nonrejection | unfailed |
| GSM534054 | renal allograft biopsy | Homo sapiens | total RNA | 642 | 2914 | nonrejection | failed |
| GSM534055 | renal allograft biopsy | Homo sapiens | total RNA | 599 | 571 | rejection | unfailed |
| GSM534056 | renal allograft biopsy | Homo sapiens | total RNA | 229 | 452 | nonrejection | failed |
| GSM534057 | renal allograft biopsy | Homo sapiens | total RNA | 209 | 3911 | nonrejection | unfailed |
| GSM534058 | renal allograft biopsy | Homo sapiens | total RNA | 819 | 7 | nonrejection | unfailed |
| GSM534059 | renal allograft biopsy | Homo sapiens | total RNA | 633 | 1020 | nonrejection | unfailed |
| GSM534060 | renal allograft biopsy | Homo sapiens | total RNA | 595 | 4409 | nonrejection | unfailed |
| GSM534061 | renal allograft biopsy | Homo sapiens | total RNA | 216 | 1464 | nonrejection | failed |
| GSM534062 | renal allograft biopsy | Homo sapiens | total RNA | 264 | 476 | rejection | failed |
| GSM534063 | renal allograft biopsy | Homo sapiens | total RNA | 502 | 4995 | rejection | failed |
| GSM534064 | renal allograft biopsy | Homo sapiens | total RNA | 811 | 1875 | rejection | unfailed |
| GSM534065 | renal allograft biopsy | Homo sapiens | total RNA | 596 | 772 | nonrejection | failed |
| GSM534066 | renal allograft biopsy | Homo sapiens | total RNA | 592 | 1985 | nonrejection | unfailed |
| GSM534067 | renal allograft biopsy | Homo sapiens | total RNA | 146 | 5383 | nonrejection | failed |
| GSM534068 | renal allograft biopsy | Homo sapiens | total RNA | 652 | 963 | nonrejection | unfailed |
| GSM534069 | renal allograft biopsy | Homo sapiens | total RNA | 652 | 10 | nonrejection | unfailed |
| GSM534070 | renal allograft biopsy | Homo sapiens | total RNA | 15 | 908 | rejection | failed |
| GSM534071 | renal allograft biopsy | Homo sapiens | total RNA | 650 | 23 | nonrejection | unfailed |
| GSM534072 | renal allograft biopsy | Homo sapiens | total RNA | 643 | 15 | nonrejection | unfailed |
| GSM534073 | renal allograft biopsy | Homo sapiens | total RNA | 8 | 273 | nonrejection | failed |
| GSM534074 | renal allograft biopsy | Homo sapiens | total RNA | 636 | 2045 | nonrejection | unfailed |
| GSM534075 | renal allograft biopsy | Homo sapiens | total RNA | 476 | 11453 | nonrejection | failed |
| GSM534076 | renal allograft biopsy | Homo sapiens | total RNA | 802 | 169 | nonrejection | unfailed |
| GSM534077 | renal allograft biopsy | Homo sapiens | total RNA | 797 | 174 | nonrejection | unfailed |
| GSM534078 | renal allograft biopsy | Homo sapiens | total RNA | 168 | 1249 | nonrejection | failed |
| GSM534079 | renal allograft biopsy | Homo sapiens | total RNA | 854 | 3233 | nonrejection | unfailed |
| GSM534080 | renal allograft biopsy | Homo sapiens | total RNA | 766 | 6034 | nonrejection | unfailed |
| GSM534081 | renal allograft biopsy | Homo sapiens | total RNA | 307 | 2806 | nonrejection | unfailed |
| GSM534082 | renal allograft biopsy | Homo sapiens | total RNA | 577 | 1562 | nonrejection | failed |
| GSM534083 | renal allograft biopsy | Homo sapiens | total RNA | 321 | 715 | nonrejection | failed |
| GSM534084 | renal allograft biopsy | Homo sapiens | total RNA | 613 | 8 | nonrejection | unfailed |
| GSM534085 | renal allograft biopsy | Homo sapiens | total RNA | 527 | 464 | nonrejection | unfailed |
| GSM534086 | renal allograft biopsy | Homo sapiens | total RNA | 90 | 5547 | nonrejection | failed |
| GSM534087 | renal allograft biopsy | Homo sapiens | total RNA | 606 | 1123 | rejection | unfailed |
| GSM534088 | renal allograft biopsy | Homo sapiens | total RNA | 545 | 9056 | nonrejection | unfailed |
| GSM534089 | renal allograft biopsy | Homo sapiens | total RNA | 658 | 37 | nonrejection | unfailed |
| GSM534090 | renal allograft biopsy | Homo sapiens | total RNA | 587 | 21 | nonrejection | unfailed |
| GSM534091 | renal allograft biopsy | Homo sapiens | total RNA | 81 | 10436 | nonrejection | failed |
| GSM534092 | renal allograft biopsy | Homo sapiens | total RNA | 357 | 390 | rejection | failed |
| GSM534093 | renal allograft biopsy | Homo sapiens | total RNA | 494 | 2052 | nonrejection | unfailed |
| GSM534094 | renal allograft biopsy | Homo sapiens | total RNA | 605 | 49 | rejection | unfailed |
| GSM534095 | renal allograft biopsy | Homo sapiens | total RNA | 437 | 176 | rejection | unfailed |
| GSM534096 | renal allograft biopsy | Homo sapiens | total RNA | 492 | 121 | nonrejection | unfailed |
| GSM534097 | renal allograft biopsy | Homo sapiens | total RNA | 421 | 4349 | nonrejection | unfailed |
| GSM534098 | renal allograft biopsy | Homo sapiens | total RNA | 399 | 3240 | nonrejection | unfailed |
| GSM534099 | renal allograft biopsy | Homo sapiens | total RNA | 704 | 17 | nonrejection | unfailed |
| GSM534100 | renal allograft biopsy | Homo sapiens | total RNA | 669 | 52 | nonrejection | unfailed |
| GSM534101 | renal allograft biopsy | Homo sapiens | total RNA | 606 | 115 | nonrejection | unfailed |
| GSM534102 | renal allograft biopsy | Homo sapiens | total RNA | 524 | 772 | nonrejection | unfailed |
| GSM534103 | renal allograft biopsy | Homo sapiens | total RNA | 1581 | 246 | nonrejection | unfailed |
| GSM534104 | renal allograft biopsy | Homo sapiens | total RNA | 1560 | 267 | nonrejection | unfailed |
| GSM534105 | renal allograft biopsy | Homo sapiens | total RNA | 551 | 770 | nonrejection | unfailed |
| GSM534106 | renal allograft biopsy | Homo sapiens | total RNA | 557 | 8 | rejection | unfailed |
| GSM534107 | renal allograft biopsy | Homo sapiens | total RNA | 547 | 18 | nonrejection | unfailed |
| GSM534108 | renal allograft biopsy | Homo sapiens | total RNA | 238 | 360 | rejection | failed |
| GSM534109 | renal allograft biopsy | Homo sapiens | total RNA | 226 | 372 | rejection | failed |
| GSM534110 | renal allograft biopsy | Homo sapiens | total RNA | 171 | 427 | rejection | failed |
| GSM534111 | renal allograft biopsy | Homo sapiens | total RNA | 105 | 493 | nonrejection | failed |
| GSM534112 | renal allograft biopsy | Homo sapiens | total RNA | 539 | 19 | rejection | unfailed |
| GSM534113 | renal allograft biopsy | Homo sapiens | total RNA | 697 | 1402 | nonrejection | unfailed |
| GSM534114 | renal allograft biopsy | Homo sapiens | total RNA | 779 | 1667 | nonrejection | unfailed |
| GSM534115 | renal allograft biopsy | Homo sapiens | total RNA | 523 | 3614 | rejection | unfailed |
| GSM534116 | renal allograft biopsy | Homo sapiens | total RNA | 39 | 1170 | nonrejection | unfailed |
| GSM534117 | renal allograft biopsy | Homo sapiens | total RNA | 113 | 1082 | nonrejection | unfailed |
| GSM534118 | renal allograft biopsy | Homo sapiens | total RNA | 833 | 513 | nonrejection | unfailed |
| GSM534119 | renal allograft biopsy | Homo sapiens | total RNA | 454 | 588 | nonrejection | unfailed |
| GSM534120 | renal allograft biopsy | Homo sapiens | total RNA | 701 | 687 | rejection | unfailed |
| GSM534121 | renal allograft biopsy | Homo sapiens | total RNA | 1411 | 2224 | rejection | unfailed |
| GSM534122 | renal allograft biopsy | Homo sapiens | total RNA | 1435 | 2350 | nonrejection | unfailed |
| GSM534123 | renal allograft biopsy | Homo sapiens | total RNA | 656 | 1317 | nonrejection | unfailed |
| GSM534124 | renal allograft biopsy | Homo sapiens | total RNA | 511 | 1462 | nonrejection | unfailed |
| GSM534125 | renal allograft biopsy | Homo sapiens | total RNA | 664 | 995 | nonrejection | unfailed |
| GSM534126 | renal allograft biopsy | Homo sapiens | total RNA | 362 | 4974 | nonrejection | unfailed |
| GSM534127 | renal allograft biopsy | Homo sapiens | total RNA | 392 | 474 | nonrejection | unfailed |
| GSM534128 | renal allograft biopsy | Homo sapiens | total RNA | 1355 | 185 | nonrejection | unfailed |
| GSM534129 | renal allograft biopsy | Homo sapiens | total RNA | 1097 | 443 | nonrejection | unfailed |
| GSM534130 | renal allograft biopsy | Homo sapiens | total RNA | 673 | 867 | rejection | unfailed |
| GSM534131 | renal allograft biopsy | Homo sapiens | total RNA | 435 | 996 | nonrejection | unfailed |
| GSM534132 | renal allograft biopsy | Homo sapiens | total RNA | 1602 | 1732 | nonrejection | unfailed |
| GSM534133 | renal allograft biopsy | Homo sapiens | total RNA | 477 | 622 | nonrejection | unfailed |
| GSM534134 | renal allograft biopsy | Homo sapiens | total RNA | 259 | 3099 | nonrejection | unfailed |
| GSM534135 | renal allograft biopsy | Homo sapiens | total RNA | 658 | 461 | nonrejection | unfailed |
| GSM534136 | renal allograft biopsy | Homo sapiens | total RNA | 466 | 4174 | nonrejection | unfailed |
| GSM534137 | renal allograft biopsy | Homo sapiens | total RNA | 543 | 46 | nonrejection | unfailed |
| GSM534138 | renal allograft biopsy | Homo sapiens | total RNA | 282 | 5181 | nonrejection | unfailed |
| GSM534139 | renal allograft biopsy | Homo sapiens | total RNA | 1612 | 11 | nonrejection | unfailed |
| GSM534140 | renal allograft biopsy | Homo sapiens | total RNA | 1480 | 1665 | nonrejection | unfailed |
| GSM534141 | renal allograft biopsy | Homo sapiens | total RNA | 639 | 2108 | nonrejection | unfailed |
| GSM534142 | renal allograft biopsy | Homo sapiens | total RNA | 638 | 498 | nonrejection | unfailed |
| GSM534143 | renal allograft biopsy | Homo sapiens | total RNA | 1497 | 8 | nonrejection | unfailed |
| GSM534144 | renal allograft biopsy | Homo sapiens | total RNA | 1475 | 30 | nonrejection | unfailed |
| GSM534145 | renal allograft biopsy | Homo sapiens | total RNA | 1440 | 65 | nonrejection | unfailed |
| GSM534146 | renal allograft biopsy | Homo sapiens | total RNA | 22 | 408 | nonrejection | unfailed |
| GSM534147 | renal allograft biopsy | Homo sapiens | total RNA | 427 | 1583 | nonrejection | unfailed |
| GSM534148 | renal allograft biopsy | Homo sapiens | total RNA | 442 | 637 | nonrejection | unfailed |
| GSM534149 | renal allograft biopsy | Homo sapiens | total RNA | 566 | 18 | nonrejection | unfailed |
| GSM534150 | renal allograft biopsy | Homo sapiens | total RNA | 353 | 947 | nonrejection | unfailed |
| GSM534151 | renal allograft biopsy | Homo sapiens | total RNA | 310 | 601 | nonrejection | unfailed |
| GSM534152 | renal allograft biopsy | Homo sapiens | total RNA | 379 | 8419 | rejection | unfailed |
| GSM534153 | renal allograft biopsy | Homo sapiens | total RNA | 323 | 5065 | nonrejection | unfailed |
| GSM534154 | renal allograft biopsy | Homo sapiens | total RNA | 516 | 793 | rejection | unfailed |
| GSM534155 | renal allograft biopsy | Homo sapiens | total RNA | 411 | 898 | nonrejection | unfailed |
| GSM534156 | renal allograft biopsy | Homo sapiens | total RNA | 361 | 4076 | nonrejection | unfailed |
| GSM534157 | renal allograft biopsy | Homo sapiens | total RNA | 225 | 3233 | nonrejection | failed |
| GSM534158 | renal allograft biopsy | Homo sapiens | total RNA | 341 | 3499 | rejection | unfailed |
| GSM534159 | renal allograft biopsy | Homo sapiens | total RNA | 583 | 7649 | nonrejection | unfailed |
| GSM534160 | renal allograft biopsy | Homo sapiens | total RNA | 623 | 1029 | nonrejection | failed |
| GSM534161 | renal allograft biopsy | Homo sapiens | total RNA | 155 | 1497 | nonrejection | failed |
| GSM534162 | renal allograft biopsy | Homo sapiens | total RNA | 1588 | 762 | rejection | unfailed |
| GSM534163 | renal allograft biopsy | Homo sapiens | total RNA | 1574 | 14 | nonrejection | unfailed |
| GSM534164 | renal allograft biopsy | Homo sapiens | total RNA | 1302 | 26 | nonrejection | unfailed |
| GSM534165 | renal allograft biopsy | Homo sapiens | total RNA | 777 | 1259 | nonrejection | failed |
| GSM534166 | renal allograft biopsy | Homo sapiens | total RNA | 1505 | 718 | nonrejection | unfailed |
| GSM534167 | renal allograft biopsy | Homo sapiens | total RNA | 1502 | 121 | nonrejection | unfailed |
| GSM534168 | renal allograft biopsy | Homo sapiens | total RNA | 1531 | 23 | nonrejection | unfailed |
| GSM534169 | renal allograft biopsy | Homo sapiens | total RNA | 1068 | 249 | nonrejection | unfailed |
| GSM534170 | renal allograft biopsy | Homo sapiens | total RNA | 1439 | 556 | rejection | unfailed |
| GSM534171 | renal allograft biopsy | Homo sapiens | total RNA | 1389 | 606 | rejection | unfailed |
| GSM534172 | renal allograft biopsy | Homo sapiens | total RNA | 1550 | 309 | nonrejection | unfailed |
| GSM534173 | renal allograft biopsy | Homo sapiens | total RNA | 192 | 4434 | nonrejection | failed |
| GSM534174 | renal allograft biopsy | Homo sapiens | total RNA | 1045 | 1835 | rejection | failed |
| GSM534175 | renal allograft biopsy | Homo sapiens | total RNA | 954 | 1926 | rejection | failed |
| GSM534176 | renal allograft biopsy | Homo sapiens | total RNA | 1360 | 3717 | rejection | unfailed |
| GSM534177 | renal allograft biopsy | Homo sapiens | total RNA | 776 | 4203 | nonrejection | failed |
| GSM534178 | renal allograft biopsy | Homo sapiens | total RNA | 1454 | 3984 | nonrejection | unfailed |
| GSM534179 | renal allograft biopsy | Homo sapiens | total RNA | 259 | 10 | nonrejection | unfailed |
| GSM534180 | renal allograft biopsy | Homo sapiens | total RNA | 197 | 72 | rejection | unfailed |
| GSM534181 | renal allograft biopsy | Homo sapiens | total RNA | 52 | 217 | nonrejection | unfailed |
| GSM534182 | renal allograft biopsy | Homo sapiens | total RNA | 1327 | 79 | rejection | unfailed |
| GSM534183 | renal allograft biopsy | Homo sapiens | total RNA | 1288 | 118 | rejection | unfailed |
| GSM534184 | renal allograft biopsy | Homo sapiens | total RNA | 1243 | 163 | rejection | unfailed |
| GSM534185 | renal allograft biopsy | Homo sapiens | total RNA | 1179 | 227 | nonrejection | unfailed |
| GSM534186 | renal allograft biopsy | Homo sapiens | total RNA | 1251 | 78 | nonrejection | unfailed |
| GSM534187 | renal allograft biopsy | Homo sapiens | total RNA | 298 | 751 | rejection | failed |
| GSM534188 | renal allograft biopsy | Homo sapiens | total RNA | 1491 | 3683 | nonrejection | unfailed |
| GSM534189 | renal allograft biopsy | Homo sapiens | total RNA | 109 | 6 | nonrejection | unfailed |
| GSM534190 | renal allograft biopsy | Homo sapiens | total RNA | 2 | 113 | rejection | unfailed |
| GSM534191 | renal allograft biopsy | Homo sapiens | total RNA | 1152 | 254 | nonrejection | unfailed |
| GSM534192 | renal allograft biopsy | Homo sapiens | total RNA | 1393 | 13 | nonrejection | unfailed |
| GSM534193 | renal allograft biopsy | Homo sapiens | total RNA | 1165 | 241 | rejection | unfailed |
| GSM534194 | renal allograft biopsy | Homo sapiens | total RNA | 1359 | 16 | nonrejection | unfailed |
| GSM534195 | renal allograft biopsy | Homo sapiens | total RNA | 1367 | 76 | rejection | unfailed |
| GSM534196 | renal allograft biopsy | Homo sapiens | total RNA | 1322 | 91 | rejection | unfailed |
| GSM534197 | renal allograft biopsy | Homo sapiens | total RNA | 1374 | 687 | nonrejection | unfailed |
| GSM534198 | renal allograft biopsy | Homo sapiens | total RNA | 104 | 5301 | nonrejection | unfailed |
| GSM534199 | renal allograft biopsy | Homo sapiens | total RNA | 1441 | 867 | rejection | unfailed |
| GSM534200 | renal allograft biopsy | Homo sapiens | total RNA | 1283 | 42 | nonrejection | unfailed |
| GSM534201 | renal allograft biopsy | Homo sapiens | total RNA | 965 | 310 | nonrejection | unfailed |
| GSM534202 | renal allograft biopsy | Homo sapiens | total RNA | 1331 | 2822 | nonrejection | unfailed |
